# Multiomic signals associated with maternal epidemiological factors contributing to preterm birth in low- and middle-income countries

**DOI:** 10.1126/sciadv.ade7692

**Published:** 2023-05-26

**Authors:** Camilo A. Espinosa, Waqasuddin Khan, Rasheda Khanam, Sayan Das, Javairia Khalid, Jesmin Pervin, Margaret P. Kasaro, Kévin Contrepois, Alan L. Chang, Thanaphong Phongpreecha, Basil Michael, Mathew Ellenberger, Usma Mehmood, Aneeta Hotwani, Ambreen Nizar, Furqan Kabir, Ronald J. Wong, Martin Becker, Eloise Berson, Anthony Culos, Davide De Francesco, Samson Mataraso, Neal Ravindra, Melan Thuraiappah, Maria Xenochristou, Ina A. Stelzer, Ivana Marić, Arup Dutta, Rubhana Raqib, Salahuddin Ahmed, Sayedur Rahman, A. S. M. Tarik Hasan, Said M. Ali, Mohamed H. Juma, Monjur Rahman, Shaki Aktar, Saikat Deb, Joan T. Price, Paul H. Wise, Virginia D. Winn, Maurice L. Druzin, Ronald S. Gibbs, Gary L. Darmstadt, Jeffrey C. Murray, Jeffrey S. A. Stringer, Brice Gaudilliere, Michael P. Snyder, Martin S. Angst, Anisur Rahman, Abdullah H. Baqui, Fyezah Jehan, Muhammad Imran Nisar, Bellington Vwalika, Sunil Sazawal, Gary M. Shaw, David K. Stevenson, Nima Aghaeepour

**Affiliations:** ^1^ Department of Anesthesiology, Perioperative and Pain Medicine, Stanford University School of Medicine, Stanford, CA, USA.; ^2^ Department of Pediatrics, Stanford University School of Medicine, Stanford, CA, USA.; ^3^ Department of Biomedical Data Science, Stanford University School of Medicine, Stanford, CA, USA.; ^4^ Department of Pediatrics and Child Health, Faculty of Health Sciences, Medical College, The Aga Khan University, Karachi, Pakistan.; ^5^ Department of International Health, Johns Hopkins Bloomberg School of Public Health, Baltimore, MD, USA.; ^6^ Centre for Public Health Kinetics, New Delhi, Delhi, India.; ^7^ Maternal and Child Health Division, International Centre for Diarrhoeal Disease Research, Dhaka, Bangladesh.; ^8^ University of North Carolina Global Projects Zambia, Lusaka, Zambia.; ^9^ Department of Obstetrics and Gynecology, University of North Carolina School of Medicine, Chapel Hill, NC, USA.; ^10^ Department of Genetics, Stanford University School of Medicine, Stanford, CA, USA.; ^11^ Department of Pathology, Stanford University School of Medicine, Stanford, CA, USA.; ^12^ Department of Computer Science, Columbia University, New York, NY, USA.; ^13^ International Centre for Diarrhoeal Disease Research, Dhaka, Bangladesh.; ^14^ Projahnmo Research Foundation, Dhaka, Bangladesh.; ^15^ Public Health Laboratory—Ivo de Carneri, Pemba, Zanzibar, Tanzania.; ^16^ Department of Obstetrics and Gynaecology, University of Zambia School of Medicine, Lusaka, Zambia.; ^17^ Department of Obstetrics and Gynecology, Stanford University School of Medicine, Stanford, CA, USA.; ^18^ Department of Pediatrics, University of Iowa, Iowa City, IA, USA.; ^19^ Johns Hopkins Bloomberg School of Public Health, Johns Hopkins University, Baltimore, MD, USA.

## Abstract

Preterm birth (PTB) is the leading cause of death in children under five, yet comprehensive studies are hindered by its multiple complex etiologies. Epidemiological associations between PTB and maternal characteristics have been previously described. This work used multiomic profiling and multivariate modeling to investigate the biological signatures of these characteristics. Maternal covariates were collected during pregnancy from 13,841 pregnant women across five sites. Plasma samples from 231 participants were analyzed to generate proteomic, metabolomic, and lipidomic datasets. Machine learning models showed robust performance for the prediction of PTB (AUROC = 0.70), time-to-delivery (*r* = 0.65), maternal age (*r* = 0.59), gravidity (*r* = 0.56), and BMI (*r* = 0.81). Time-to-delivery biological correlates included fetal-associated proteins (e.g., ALPP, AFP, and PGF) and immune proteins (e.g., PD-L1, CCL28, and LIFR). Maternal age negatively correlated with collagen COL9A1, gravidity with endothelial NOS and inflammatory chemokine CXCL13, and BMI with leptin and structural protein FABP4. These results provide an integrated view of epidemiological factors associated with PTB and identify biological signatures of clinical covariates affecting this disease.

## Teaser

Deep biological profiling and machine learning reveal insights into the epidemiology of preterm birth.

## INTRODUCTION

Preterm birth (PTB)—birth before 37 weeks of gestation—is the leading cause of mortality and morbidity in children under 5 years of age across the globe, with a particularly high prevalence in low- and middle-income countries (LMICs) (*1*). Children born preterm are at increased risk of multiple life-threatening short-term complications and long-term neurological, cardiovascular, and metabolic morbidities (*2*). PTB therefore affects the lives of the mother, child, and family and poses a high burden to global public health that falls overwhelmingly on the health systems of LMICs (*3*). However, efforts to improve our understanding of PTB are hindered by its complex etiologies. Proposed mechanisms for spontaneous PTB include infection, inflammation, loss of maternal-fetal immune tolerance, placental senescence, cervical insufficiency, and vascular disease, with each mechanism possibly affecting different populations to varying degrees (*4*–*6*). The complexity of PTB has also led to a scarcity of possible interventions and screening procedures, underscoring the need to further the understanding of this disease (*7*).

Epidemiologic associations between PTB and maternal clinical history, demographic characteristics, and social determinants have been extensively described, ​​but have been limited in inference owing to a lack of attendant biological data (*8*). For example, PTB has a well-known association with maternal obstetric history. In particular, history of a previous PTB remains among the strongest risk factors for PTB in an active pregnancy (*9*), although short interpregnancy intervals (*10*) and histories of stillbirth (*11*), Cesarean delivery (*12*), and other previous adverse pregnancy outcomes all contribute to increased risk of PTB (*9*, *13*, *14*). Socioeconomic determinants of health have recently come into focus as key players that affect the outcome of gestation (*14*). Environmental exposures (*15*), quality and number of antenatal care visits (*16*, *17*), psychological distress levels (*18*, *19*), and experiences of racial bias (*20*) may also influence the health of a woman’s pregnancy. Furthermore, these maternal characteristics intersect and interact in complex ways that may further compound PTB risks (*15*, *21*).

The recent development of high-throughput technologies to profile different levels of an individual’s biology in health and disease has brought forth a new era of precision medicine (*14*). By using high-dimensional measurements of an individual’s biological systems—such as their transcripts (transcriptome), proteins (proteome), metabolites (metabolome), and immune cell frequencies and functions (immunome)—biomarkers of disease can be found and mechanistic hypotheses on biological processes can be inferred (*4*, *5*). In the context of pregnancy, multiomic profiling, together with the development and application of appropriate machine learning frameworks to interpret these complex data, has led to a better understanding of the immunologic and proteomic changes that regulate the chronicity of pregnancy (*22*–*24*) and the cross-talk between biological modalities that determines the onset of labor (*25*). Furthermore, this approach has previously clarified the biology of adverse pregnancy outcomes (*26*–*28*), underscoring its potential for improving our understanding of PTB. However, population-specific differences in the biological processes that precede pregnancy complications and the presence of other possible confounding factors have limited the generalizability of multiomic models (*29*).

Here, we leveraged a large multinational cohort of pregnant women across four LMICs to evaluate a set of epidemiologically derived maternal factors associated with PTB. We used proteomic, metabolomic, and lipidomic profiling and performed multivariate modeling in a subcohort of these women to investigate the biological correlates of some of these maternal covariates for their underlying association with PTB. By combining epidemiological data from the full cohort and biological insights from the multiomic data of a subcohort, we built an integrated view of how epidemiological associations with PTB can affect maternal biologic measures. Our approach simultaneously identified signatures for maternal covariates affecting PTB and allowed us to propose an innovative conceptual framework for studying the connections between population-level risk factors and underlying pathological mechanisms of PTB.

## RESULTS

### Participants and study design

**Fig. 1.**
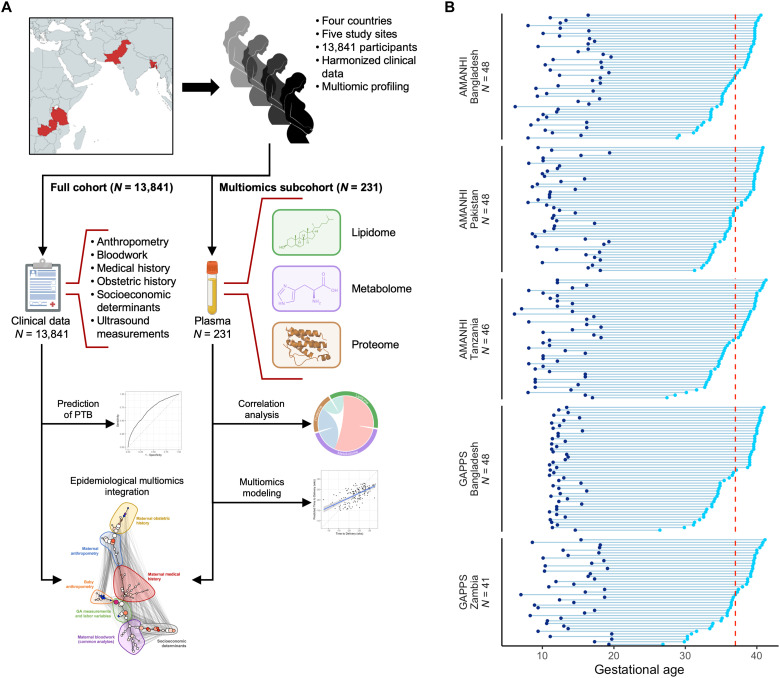
Study overview. (**A**) Maternal clinical data and plasma samples were collected from a cohort of 13,841 pregnant women across five sites in four low- and middle-income countries (LMICs). Plasma samples taken during early and mid pregnancy from a subcohort of 231 of these women were further analyzed to generate targeted lipidomic, untargeted metabolomic, and targeted proteomic datasets. Clinical data from the full cohort were used for the prediction of preterm birth (PTB). Multiomic profiling data from the multiomics subcohort were used for interomic correlation analysis and for the prediction of maternal clinical covariates. Clinical data from the full cohort and multiomic data from the multiomics subcohort were used for the epidemiological multiomic integration. (**B**) Raster plot depicting the gestational age (GA) at sampling for each woman in the multiomics subcohort stratified by site of origin, where each line represents an individual woman and the dark blue circles and light blue circles represent sampling dates and delivery dates, respectively. The dashed red line at 37 weeks of GA indicates the boundary between preterm (PTB) and term births.

Maternal covariates and plasma samples were collected during early to mid pregnancy from a multinational cohort of 13,841 pregnant women recruited by the Alliance for Maternal and Newborn Health Improvement (AMANHI) and the Global Alliance to Prevent Prematurity and Stillbirth (GAPPS) (Fig. 1A). AMANHI sites included Sylhet, Bangladesh (AMANHIB); Karachi, Pakistan (AMANHIP); and Pemba, Tanzania (AMANHIT); while GAPPS sites included Matlab, Bangladesh (GAPPSB) and Lusaka, Zambia (GAPPSZ). Of the 13,841 pregnancies, 1578 (11.4%) resulted in PTBs (defined here as delivery before 37 gestational weeks). Maternal covariates included maternal characteristics previously associated with PTB—e.g., weight and height—as well as maternal clinical history, demographics, and socioeconomic determinants. Participant demographics, antepartum parameters, and pregnancy characteristics are listed in Table 1. A full list of the maternal covariates measured can be found in the Supplementary Materials.

**Table 1.** Full cohort and multiomics subcohort characteristics. Summary of the demographic, clinical, and other participant characteristics for the cohorts in the study.

Maternal characteristics	Full cohort (*N* = 13,841)		Multiomics subcohort (*N* = 231)	
	Percentage or median [interquartile range]		Percentage or median [interquartile range]	
Age (years)	13,815	25 [22–30]	229	25 [22–29]
Body mass index (kg/m^2^)	13,200	22.0 [19.8–25.3]	226	22.0 [19.9–25.3]
Gravidity	13,831	3 [1–4]	231	3 [1–4]
Parity (% nulliparous)	13,831	3,905 (28.2%)	231	70 (30.3%)
Level of education (years)	13,816	8 [5–10]	231	8 [5–10]
Pregnancy characteristics				
Gestational age at delivery (weeks)	13,841	39.3 [38.1–40.1]	231	37.1 [34.4–39.6]
Preterm delivery (<37 weeks)	13,841	1,578 (11.4%)	231	113 (48.9%)
Infant sex	13,620	Female (48.6%)	231	Female (53.6%)
Birthweight (g)	11,985	2,970 [2,605–3,300]	213	2,575 [2,210–3,025]
Spontaneous delivery	13,555	13,555 (79.9%)	229	224 (97.8%)
Site				
Sylhet, Bangladesh (AMANHIB)	13,841	2,845 (20.6%)	231	48 (20.8%)
Karachi, Pakistan (AMANHIP)	13,841	2,335 (16.9%)	231	48 (20.8%)
Pemba, Tanzania (AMANHIT)	13,841	4,115 (29.7%)	231	46 (19.9%)
Matlab, Bangladesh (GAPPSB)	13,841	3,434 (24.8%)	231	48 (20.8%)
Lusaka, Zambia (GAPPSZ)	13,841	1,112 (8.0%)	231	41 (17.7%)
Comorbidities				
History of preterm birth	10,001	8.4%	161	18.6%
History of stillbirth	10,207	8.7%	165	8.5%
History of miscarriage	10,317	23.0%	165	25.5%
History of cesarean	10,169	9.6%	163	4.3%
Hypertension	13,450	3.0%	228	4.8%
Diabetes	13,435	0.5%	227	0.4%

### Measurement of the maternal proteome, metabolome, and lipidome in early and mid pregnancy

**Fig. 2.**
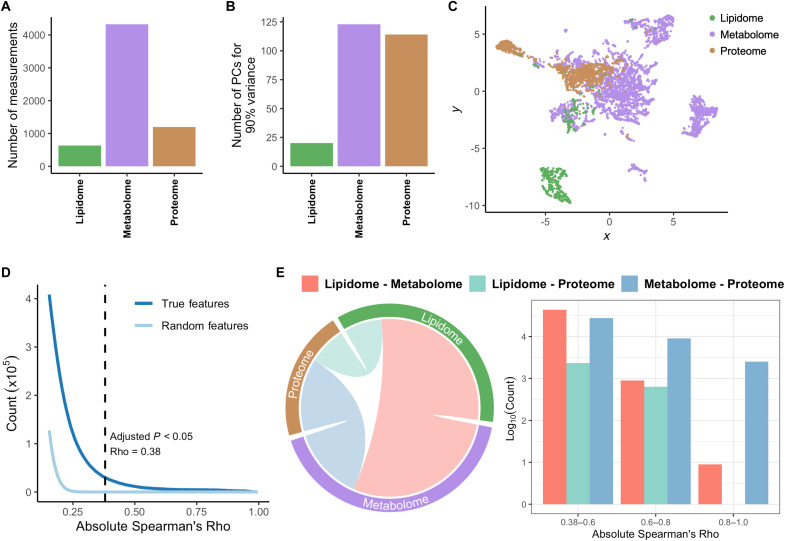
Multiomic characterization of early and mid pregnancy. Plasma samples taken during early and mid pregnancy from a subcohort of 231 women were analyzed to generate targeted lipidomic, untargeted metabolomic, and targeted proteomic datasets. (**A**) Quantification of the number of measurements (features) of each different omic analyzed. (**B**) Estimation of the modularity—i.e., the degree of internal correlation between features in a given omic dataset—using the number of principal components needed to explain 90% of the variance. (**C**) A two-dimensional representation of the multiomic correlation space was generated by first calculating the correlation matrix of the feature space and then using the UMAP dimensionality reduction algorithm for visualization, where green, purple, and brown circles represent lipidomic, metabolomic, and proteomic features, respectively. (**D** and **E**) Interomic and intraomic Spearman correlations were quantified and assessed for significance using a cutoff value of Bonferroni-adjusted Spearman *P* < 0.05, which corresponded to an absolute Spearman Rho > 0.38. (D) Distribution of all correlations by absolute strength of association for the multiomic dataset (dark blue) and a random dataset (light blue), where each simulated feature was generated through bootstrapping of the true feature distribution. (E) Visualization of significant interomic correlations. Left: Chord diagram showing the relative distribution of significant interomic correlations, where the outer ring depicts each individual omic and links correspond to interomic correlations, with colors assigned as shown in the legend. The size of the links is proportional to the total number of significant interactions normalized to the total number of possible correlations between each omic pair. Right: Quantification of the number of significant weak (0.38 to 0.6), moderate (0.6 to 0.8), and strong (0.8 to 1.0) absolute correlations between each omic pair.

Plasma samples taken in early to mid pregnancy [median gestational age (GA) at sampling = 12.2 weeks; SD = 3.2 weeks] from 231 participants were further analyzed to generate proteomic, metabolomic, and lipidomic datasets (Fig. 1A). This subcohort was chosen in a case-control design and included 113 pregnant women with a PTB (48.9%) and 118 with a term delivery (51.1%). Samples were collected from singleton pregnancies without congenital malformations and with spontaneous live deliveries. Case and control samples were matched within each site based on GA at sampling, and maternal covariates were not significantly different between cases and controls. The individual variability in the timing of each visit and eventual delivery enabled the creation of a continuous variable for time at sampling (GA at sampling) and for birth (GA at birth) (Fig. 1B). The targeted multiplexed proteomic immunoassay measured 1161 proteins using 1196 probes, while the untargeted metabolomic and targeted lipidomic produced 4329 and 632 measurements, respectively, for a total of 6157 multiomic features (Fig. 2A). Dataset modularity, as estimated by the number of principal components needed to explain 90% of the variance, indicated a similar information content in the proteomic and metabolomic assays despite their difference in size, with a lower modularity in the lipidomic data (Fig. 2B). The combined multiomic dataset was visualized by first calculating the Spearman correlation between each pair of features and then creating a two-dimensional representation of the feature correlation space using the Uniform Manifold Approximation and Projection (UMAP) algorithm (Fig. 2C) (*30*).

The distribution of significant correlations within (intra-) and between (inter-) omics in pregnancy revealed the rigorous orchestration of biological processes during gestation (Fig. 2, D and E). Given the different scales and statistical distribution properties of each biological modality, the absolute nonparametric Spearman correlation coefficient was used to quantify the strength of the associations between features. After adjusting for multiple hypothesis testing, 540,653 of all 18,951,246 feature pairs (2.9%) were significantly correlated (Bonferroni-adjusted Spearman *P* < 0.05, |Spearman Rho| > 0.38) (Fig. 2D). Significant correlations were driven by robust intraomic coordination so that associations within individual omics accounted for 84% of all significant correlations observed. Nonbiological sources of bias, such as technical bias introduced during data generation, can lead to correlation structures within each omic. As such, interomic correlations can more faithfully reflect real biological concordance. After normalizing for the size of each modality, interactions between the lipidome and the metabolome emerged as the largest relative contributor to interomic correlations, accounting for 52% of all significant interomic associations (Fig. 2E).

The early and mid pregnancy interactome consisted of 73,714 weak (|Rho| = 0.38 to 0.6), 10,585 moderate (|Rho| = 0.6 to 0.8), and 2549 strong (|Rho| = 0.8 to 1.0) interomic correlations, with strong interactions almost exclusively driven by associations between metabolome and proteome features (Fig. 2E). Gene set overrepresentation analysis for Gene Ontology (GO) terms (*31*, *32*) performed on the proteins with strong correlations with metabolomic features revealed significant enrichment of pathways associated with cell cycle and cellular metabolism (fig. S1A). Pathway analysis (*33*) performed on the set of metabolites strongly correlated with proteomic features indicated the enrichment of pathways associated with amino acid metabolism (fig. S1B). These results suggest that the strongest interomic correlations were driven by proteins and metabolites with interconnected metabolic functions. Overall, the correlations found indicate a strong alignment between the different biological systems profiled, which greatly surpasses what would be expected by random chance. Thus, the pregnancy interactome highlights the strict regulation of these biological systems and their cross-talk during gestation.

### Epidemiological factors—Maternal covariates and PTB

**Fig. 3.**
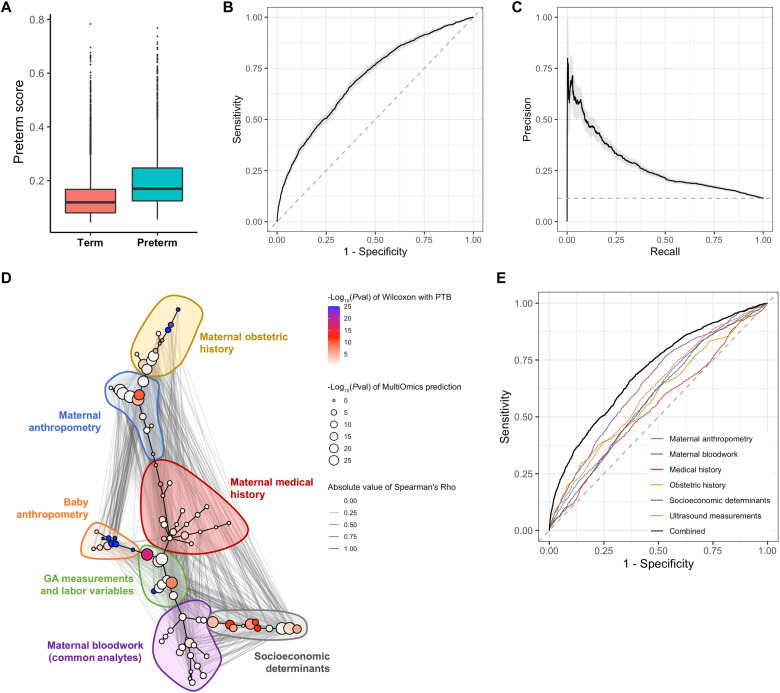
An epidemiological model for the prediction of PTB. A cross-validated gradient-boosted tree (XGBoost) model for the prediction of PTB was trained on the epidemiological data of the full cohort of 13,841 pregnant women. (**A**) Distribution of the cross-validated preterm risk scores—the probability PTB assigned by the epidemiological model to each participant—stratified by PTB status (*P* = 6.1 × 10^−147^, *N* = 13,841). (**B**) Receiver operating characteristic (ROC) curve for the epidemiological model [area under the ROC curve (AUROC) = 0.70, 95% CI: 0.69 to 0.71]. (**C**) Precision-recall curve (PRC) for the epidemiological model [area under the PRC (AUPRC) = 0.27, 95% CI: 0.25 to 0.29]. (**D**) Correlation network of the maternal and fetal covariates collected during the study, where nodes are grouped by clinical categories and were arranged into a two-dimensional layout using a minimum spanning tree. Each node represents a different covariate, where the color of the node represents the strength of the univariate association of the covariate with PTB and the size of the node represents the predictability of the covariate using multiomic data from the multiomics subcohort. Edges were drawn between significantly associated covariates after adjusting for multiple hypothesis testing, and edge thickness represents the strength of the Spearman correlation between the two covariates. (**E**) A cross-validated XGBoost model was built for the prediction of PTB on the full cohort of women using only the covariates from each of the clinical categories used, and the performance of each model was visualized using an ROC curve. See the Supplementary Materials for more details on the clinical covariates included in each category. The gray shadows in the ROC curve and PRC plots represent the 95% CI of the respective curves.

Epidemiological characteristics associated with PTB have been previously described (*3*, *34*). To investigate these associations in the full cohort with a multivariate approach, a cross-validated gradient-boosted tree model (XGBoost) (*35*) was built using only the maternal covariates measured in antenatal visits before 28 weeks and was able to distinguish between PTB and term births (Wilcoxon rank sum test *P* = 6.1 × 10^−147^, *N* = 13,841) (Fig. 3A). The model used a repeated cross-validation scheme to get an unbiased estimate of the performance of the model for unseen participants (Materials and Methods). The area under the receiver operating characteristic curve (AUROC) for the model was 0.70 [95% confidence interval (CI), 0.69 to 0.71], highlighting the difficulty of the predictive task (Fig. 3B and fig. S2A). Given the class imbalance in the complete cohort, the area under the precision-recall curve (AUPRC) was also used to assess the performance of the model (*36*), with an AUPRC of 0.27 (95% CI, 0.25 to 0.29) (Fig. 3C and fig. S2B). When adjusted for the prevalence of PTB in this population, the relative AUPRC, or Lift, of the model showed a 2.4 improvement over the baseline. The performance of the model built only on maternal covariates demonstrated the existence of a potential signature for PTB risk in this population.

The maternal covariates and measurements of the growing fetus taken during pregnancy and at birth formed a correlation network, which was visualized using a minimum spanning tree (Fig. 3D and fig. S3). The interconnectedness of maternal covariates within and across the clinical categories considered (e.g., maternal obstetric history and socioeconomic determinants; see the Supplementary Materials for more details) underscored the importance of a holistic view of maternal and fetal health when studying PTB. Top features associated with PTB included whether the pregnancy had multiple fetuses (Bonferroni-adjusted Wilcoxon rank sum test *P* = 3.0 × 10^−72^, *N* = 13,841), the mother’s history of previous PTB (Bonferroni-adjusted Wilcoxon rank sum test *P* = 1.1 × 10^−26^, *N* = 10,001), and the mother’s history of previous Cesarean delivery (Bonferroni-adjusted Wilcoxon rank sum test *P* = 8.2 × 10^−25^, *N* = 10,169).

Given the importance of maternal obstetric history for the prediction of PTB in the full cohort, the role of these features was further characterized in parous and nulliparous participants separately. To quantify the impact of including obstetric history when predicting PTB in these populations, models were built with and without these features in the nulliparous, parous, and combined populations and their performances were compared (fig. S4). As expected, inclusion of the maternal obstetric history did not change the performance of the model in nulliparous participants, while the performance of the model in parous participants markedly improved when these features were included. Furthermore, training on the full cohort with or without the obstetric history features led to an overall improvement in the performance of the model, demonstrating the benefit to model performance of a large and heterogeneous population.

For a deeper assessment of the importance of each of these facets of maternal and fetal health in the prediction of PTB, cross-validated XGBoost models using only the features associated with each of these facets were built. The performance of each of these models was assessed using AUROC and AUPRC and is shown in Table 2. Notably, the maternal bloodwork module—consisting of common clinical analytes measured during pregnancy as part of routine antenatal care (see the Supplementary Materials for full list of analytes)—was the clinical category with the highest AUROC, while the model built using the maternal nonobstetric medical history had the worst performance (Fig. 3E). Information from all clinical categories was necessary to obtain the best performance.

**Table 2.** Performance statistics of the model built on each clinical category for the prediction of PTB. Machine learning–based cross-validated models were built on the full study cohort for the prediction of PTB using epidemiological features. Model performances were assessed using the area under the receiver operating characteristic curve (AUROC), the area under the precision-recall curve (AUPRC), and the Wilcoxon rank sum test. See the Supplementary Materials for more details on the clinical covariates included in each category.

Clinical category	AUROC [95% CI]	AUPRC	Wilcoxon rank sum test *P*
Maternal anthropometry	0.59 [0.58–0.61]	0.16 [0.15–0.17]	9.8 × 10^−33^
Maternal bloodwork	0.65 [0.63–0.66]	0.19 [0.18–0.21]	9.3 × 10^−82^
Medical history	0.54 [0.53–0.56]	0.13 [0.13–0.14]	8.9 × 10^−8^
Obstetric history	0.59 [0.57–0.60]	0.17 [0.16–0.18]	1.1 × 10^−26^
Socioeconomic determinants	0.58 [0.57–0.60]	0.14 [0.14–0.15]	3.9 × 10^−26^
Ultrasound measurements	0.61 [0.60–0.63]	0.19 [0.18–0.21]	3.1 × 10^−48^

The correlation network was also used to visualize the impact of each covariate on maternal omics. This impact was estimated with the covariate’s predictability by multivariate models built with multiomic data in the subcohort for which such data were available (Fig. 3D). Highly predictable covariates clustered together in the MST layout, with the strongest biological associations arising from covariates in the maternal anthropometry, maternal obstetric history, GA measurements and labor variable, and socioeconomic determinant modules. In summary, this analysis described a multivariate risk factor association of PTB (conserved across multiple geographic regions) and revealed which early and mid pregnancy measurements were most useful for predicting PTB.

### Multiomic modeling of the maternal proteome, metabolome, and lipidome predicts time-to-delivery

**Fig. 4.**
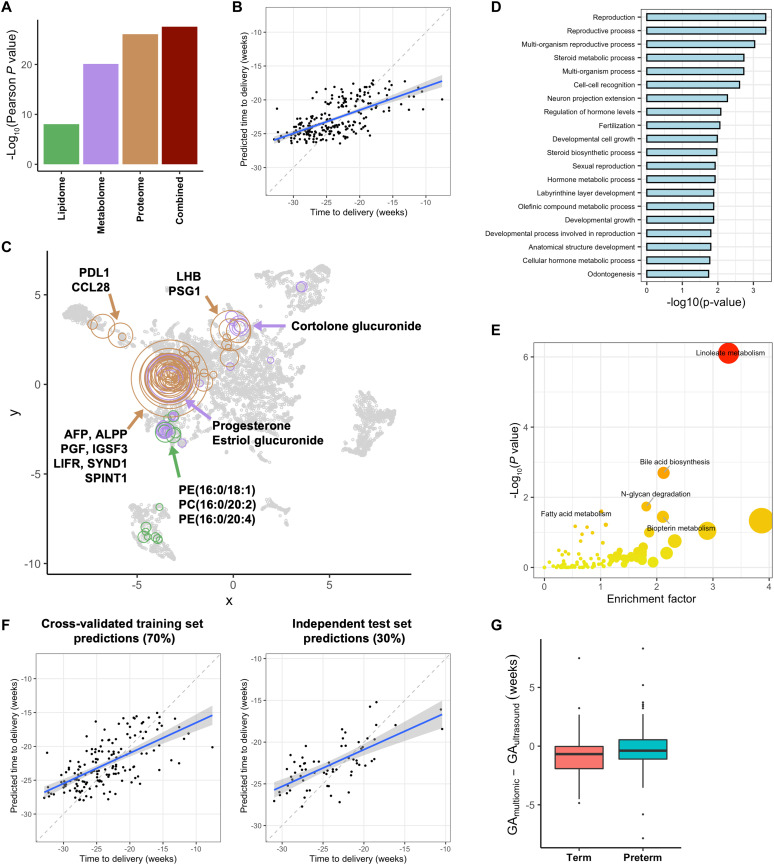
Multiomic modeling of the maternal proteome, metabolome, and lipidome predicts time-to-delivery. A cross-validated gradient-boosted tree (XGBoost) model for the prediction of time from sampling to delivery was trained on each individual omic as well as the combined multiomic dataset. (**A**) Comparison of the cross-validated performance of each model for the prediction of time-to-delivery. (**B**) Time-to-delivery predictions of the combined multiomic model (Pearson’s *r* = 0.65, 95% CI: 0.57 to 0.72, *P* = 2.6 × 10^−28^, RMSE = 3.7 weeks, MAE = 3.0 weeks, *N* = 226). (**C**) Two-dimensional UMAP visualization of the multiomic features significantly correlated with time-to-delivery. Each circle represents a feature, with sizes proportional to Spearman correlation with time-to-delivery, and colors representing modality. (**D**) Gene Ontology (GO) overrepresentation analysis performed on the plasma proteomic features significantly correlated with time-to-delivery as assessed using Fisher’s exact test. (**E**) Metabolic pathway enrichment analysis performed on the metabolomic features significantly correlated with time-to-delivery. (**F**) Participants were randomly split into a training set (*N* = 159, 70%) and a test set (*N* = 67, 30%) to build a minimal XGBoost model for the prediction of time-to-delivery. Left: Cross-validated model predictions in the training set (Pearson’s *r* = 0.64, 95% CI: 0.54 to 0.73, *P* = 7.4 × 10^−20^, RMSE = 3.9 weeks, MAE = 3.1 weeks, *N* = 159). Right: Model predictions in the test set (Pearson’s *r* = 0.68, 95% CI: 0.53 to 0.79, *P* = 1.7 × 10^−10^, RMSE = 3.4 weeks, MAE = 2.8 weeks, *N* = 67). (**G**) A cross-validated XGBoost model for the prediction of GA at sampling was trained on the combined multiomic dataset. Boxplot depicts the discrepancy between predicted GA and ultrasound GA stratified by PTB status. The blue lines and blue shadows in scatterplots represent the regression line and 95% CI of the predicted values versus the ground truth.

The progression of pregnancy and the onset of labor are highly coordinated biological processes that can be measured using multiomic profiling (*5*, *22*–*25*, *37*). To determine whether biological signatures were present in the multiomics subcohort that could capture the time from sampling to delivery, a cross-validated XGBoost model was built with the integrated maternal proteome, metabolome, and lipidome during early and mid pregnancy and strongly predicted the time-to-delivery [Pearson’s *r* (*r*) = 0.65, 95% CI: 0.57 to 0.72, *P* = 2.6 × 10^−28^, root mean square error (RMSE) = 3.7 weeks, mean absolute error (MAE) = 3.0 weeks, *N* = 226] (Fig. 4, A and B). Models built on each omic dataset individually had statistically significant performance in predicting time-to-delivery, with the proteome (*P* = 8.5 × 10^−27^, *r* = 0.63) having the highest predictive power—matching that of the integrated model—followed by the metabolome (*P* = 7.5 × 10^−21^, *r* = 0.57) and the lipidome (*P* = 9.6 × 10^−9^, *r* = 0.37) (Fig. 4A).

Understanding how the model’s error distributes among classes and the range of target values is important for its interpretability and application. The multiomic model for the time from sampling to delivery underestimates the time-to-delivery when far from delivery, while it slightly overestimates the time-to-delivery when closer to delivery (fig. S5A). Consequently, the model generally overestimates PTB risk by underestimating the time-to-delivery in term deliveries (fig. S5B). However, when incorporating the sampling time (which is known) into the model’s time-to-delivery predictions, the model is able to discriminate between PTB and term births (Wilcoxon rank sum test *P* = 0.04, *N* = 226) (fig. S5C).

Generalizability across sites is critical for the translational potential of predictive multiomic models. However, the integrated model’s performance varied by site, with the highest performance in the AMANHI Pakistan site (*r* = 0.83) and the lowest in the GAPPS Bangladesh site (*r* = 0.41; Table 3). To better understand the impact of integrating heterogeneous sites during training, site-specific models were trained and their performance was compared to the performance of the integrated model in each site (fig. S6A). This analysis revealed that all sites benefit from training using the full cohort, with a median improvement of 0.17 in the Pearson correlation between each site’s predicted values and the ground truth. In particular, the sites that benefit most from integrated training are those where the site-specific models have poor performance, such as the GAPPS Bangladesh site, with an increase of 0.41 in the Pearson correlation of the site’s predictions to the ground truth. Similarly, assessing the performance of the model in cohorts with standard PTB rates is necessary to establish its generalizability to real-world populations. To estimate the performance of the model in a population with a PTB rate of 11.4% (the prevalence of PTB in the full cohort), random cohorts with this PTB rate were sampled from the multiomics subcohort and the model performance metrics in the sampled cohorts were calculated (fig. S6B). The model’s performance in the sampled cohorts was not significantly inferior to its performance in the multiomics subcohort, indicating its applicability to real-world populations.

**Table 3.** Performance statistics for the model for the prediction of time-to-delivery. A machine learning–based cross-validated model was built for the prediction of time-to-delivery using multiomic features. Model performance in each study site was assessed using Pearson’s correlation coefficient (Pearson’s *r*), the root mean square error (RMSE), and the mean absolute error (MAE).

Site	*N*	Pearson’s *r* [95% CI]	Pearson correlation *P*	RMSE (weeks)	MAE (weeks)
Sylhet, Bangladesh (AMANHIB)	48	0.64 [0.43–0.78]	1.1 × 10^−6^	3.2	2.6
Karachi, Pakistan (AMANHIP)	48	0.83 [0.72–0.90]	1.7 × 10^−13^	3.4	2.9
Pemba, Tanzania (AMANHIT)	43	0.52 [0.26–0.71]	3.8 × 10^−4^	4.4	3.3
Matlab, Bangladesh (GAPPSB)	48	0.41 [0.14–0.62]	3.7 × 10^−3^	3.5	3.1
Lusaka, Zambia (GAPPSZ)	39	0.66 [0.43–0.81]	4.9 × 10^−6^	4.2	3.4
Combined	226	0.65 [0.57–0.72]	1.6 × 10^−29^	3.7	3.0

Features significantly associated with the time-to-delivery included some that have been previously described in the literature as well as proteins and metabolites correlated to the chronicity of pregnancy and time-to-delivery (Fig. 4C). Pregnancy-associated steroid hormones such as estriol glucuronide (Spearman’s Rho (Rho) = 0.56) and progesterone (Rho = 0.42) clustered together with placental and fetal proteins such as placental alkaline phosphatase (ALPP; Rho = 0.67), α fetoprotein (AFP; Rho = 0.62), and placental growth factor (PGF; Rho = 0.55). Notably, immune system–associated proteins programmed death ligand 1 (PD-L1; Rho = 0.43), C-C motif chemokine ligand 28 (CCL28; Rho = 0.44), and leukemia inhibitory factor (LIF) receptor (LIFR; Rho = 0.53) had a strong positive correlation with time-to-delivery. GO term overrepresentation analysis performed on the proteins significantly associated with time-to-delivery revealed an enrichment in the reproductive process GO term (Fisher’s exact test *P* = 4.5 × 10^−4^) and the steroid hormone biosynthesis GO term (*P* = 0.002) (Fig. 4D). Pathway analysis performed on the set of metabolites significantly correlated with time-to-delivery indicated the enrichment of the linoleate metabolism pathway (*P* = 7.8 × 10^−7^) (Fig. 4E).

Predictive multiomic models that maintain high performance with a minimal number of input measurements are of particular interest for their potential impact in LMICs. To explore the possibility of a minimal model for the prediction of time-to-delivery, participants were randomly split into a training set (*N* = 159, 70%) for feature selection and a test set (*N* = 67, 30%) for independent assessment of the performance of the minimal model. The feature selection paradigm is described in Materials and Methods. The minimal model consisted of only three features and achieved the performance of the full model in the independent test set (*r* = 0.68, 95% CI: 0.53 to 0.79, *P* = 1.7 × 10^−10^, RMSE = 3.4 weeks, MAE = 2.8 weeks, *N* = 67) (Fig. 4F). The features chosen for the minimal model included proteins ALPP, AFP, and serine peptidase inhibitor Kunitz type 1 (SPINT1; Rho = 0.59) (fig. S7).

Previous work on the chronicity of pregnancy has postulated that models for predicting GA at sampling show “accelerated gestational clocks” in participants who will deliver prematurely (*24*). In the multiomics subcohort, a cross-validated XGBoost model accurately predicted GA at sampling (*r* = 0.84, 95% CI: 0.80 to 0.87, *P* = 1.4 × 10^−62^, RMSE = 1.9 weeks, MAE = 1.4 weeks, *N* = 231) (fig. S8), and the discrepancy between the multiomic GA and the ultrasound-based GA showed an accelerated gestational clock in the PTB group (*P* = 0.007, *N* = 231) (Fig. 4G). In sum, multiomic profiling robustly captures biological processes in early and mid pregnancy, which can predict time-to-delivery and further show evidence of accelerated gestational clocks in women who will deliver prematurely.

### CXCL13 and eNOS are age-independent markers of pregnancy memory

**Fig. 5.**
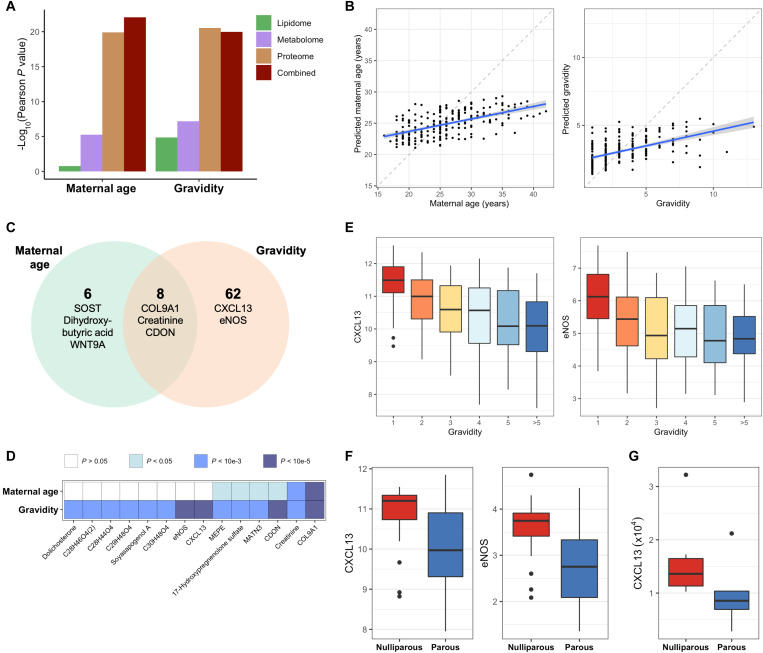
CXCL13 and eNOS are age-independent markers of pregnancy memory. Cross-validated gradient-boosted tree (XGBoost) models for the prediction of maternal age and gravidity were trained on each individual omic as well as the combined multiomic dataset. (**A**) Comparison of the cross-validated performance of each model for the prediction of maternal age and gravidity. (**B**) Left: Maternal age predictions of the combined multiomic model (Pearson’s *r* = 0.59, 95% CI: 0.50 to 0.67, *P* = 9.0 × 10^−23^, RMSE = 5.0 years, MAE = 3.8, *N* = 229). Right: Gravidity predictions of the combined multiomic model (Pearson’s *r* = 0.56, 95% CI: 0.47 to 0.64, *P* = 1.1 × 10^−20^, RMSE = 2.0, MAE = 1.5, *N* = 231). The blue line and blue shadow represent the regression line and 95% CI of the predicted values versus the ground truth. (**C**) Example features significantly associated with maternal age, gravidity, or both. (**D**) Tile chart depicting the strength of the association between the top features significantly correlated with maternal age and gravidity, where a darker color depicts a stronger association. (**E**) CXCL13 (left) and eNOS (right) levels in maternal plasma in early and mid pregnancy stratified by gravidity. (**F**) Validation of findings in plasma proteome in an independent set of early pregnancy samples in the AMANHI and GAPPS biorepositories (*N* = 70). CXCL13 (left) and eNOS (right) levels in early pregnancy stratified by nulliparity. (**G**) Validation of findings in plasma proteome in an independent cohort from Lucile Packard Children’s Hospital at Stanford (*N* = 17). CXCL13 levels in early pregnancy stratified by nulliparity.

A mother’s age and obstetric history both have an impact on her risk of PTB, yet the independent risk contribution of these intercorrelated factors to PTB is unclear (*11*, *13*, *38*). In the multiomics subcohort, maternal age and gravidity were both predictable with the integrated multiomic data (Age: *r* = 0.59, 95% CI: 0.50 to 0.67, *P* = 9.0 × 10^−23^, RMSE = 5.0 years, MAE = 3.8, *N* = 229; Gravidity: *r* = 0.56, 95% CI: 0.47 to 0.64, *P* = 1.1 × 10^−20^, RMSE = 2.0, MAE = 1.5, *N* = 231) (Fig. 5, A and B). Maternal age and gravidity shared multiple significantly correlated features, of which type IX collagen (COL9A1) had the strongest association with either covariate (Age: Bonferroni-adjusted Spearman correlation *P =* 3.1 × 10^−18^, Rho = −0.58; Gravidity: Bonferroni-adjusted Spearman correlation *P =* 1.1 × 10^−19^, Rho = −0.60) (Fig. 5C and fig. S9, A and B). This is partially explained by the positive correlation between maternal age and gravidity (*r* = 0.61 in the full cohort; *r* = 0.59 in the multiomics subcohort). Despite this positive correlation, proteome features C-X-C motif chemokine ligand 13 (CXCL13) and endothelial nitric oxide (NO) synthase (eNOS) were significantly correlated with gravidity but not significantly associated with maternal age (Fig. 5D), suggesting age-independent effects on the maternal proteome associated with the mother’s obstetric history. Other features with age-independent significant associations with maternal gravidity included soyasapogenol A and dolichosterone.

A multiple regression model for each of these features fit to maternal age and gravidity adjusted with random intercepts for site of origin confirmed that the age-independent associations between gravidity and CXCL13 and eNOS are also site independent (CXCL13: mixed-model fit *P* = 6.4 × 10^−9^; eNOS: mixed-model fit *P* = 5.8 × 10^−6^) (fig. S10). Closer interrogation of the relationship between CXCL13 and eNOS and maternal gravidity revealed inverse correlations between these two proteins and maternal gravidity during early and mid pregnancy (CXCL13: Rho = −0.46; eNOS: Rho = −0.39) (Fig. 5E). The findings regarding eNOS and CXCL13 expression were further validated in two independent cohorts—a set of early pregnancy samples from the AMANHI and GAPPS biorepositories (*27*) (*N* = 70) and a cohort from Lucile Packard Children’s Hospital at Stanford University (*37*) (*N* = 17). In the AMANHI and GAPPS cohort, nulliparous women had significantly increased levels of CXCL13 (Wilcoxon rank sum test *P* = 0.003, *N* = 70) and eNOS (Wilcoxon rank sum test *P* = 0.0003, *N* = 70) in early pregnancy when compared with women with previous pregnancies (Fig. 5F). In the Stanford cohort, nulliparous women and those with previous pregnancies had significant differences in CXCL13 expression in the first trimester (Wilcoxon rank sum test *P* = 0.008, *N* = 17), validating the results obtained in the multiomics subcohort of this study (Fig. 5G and fig. S11). Overall, these findings reveal previously unknown age-independent effects of previous pregnancies on the maternal biology during future pregnancies.

### Predictive modeling of the maternal body mass index

**Fig. 6.**
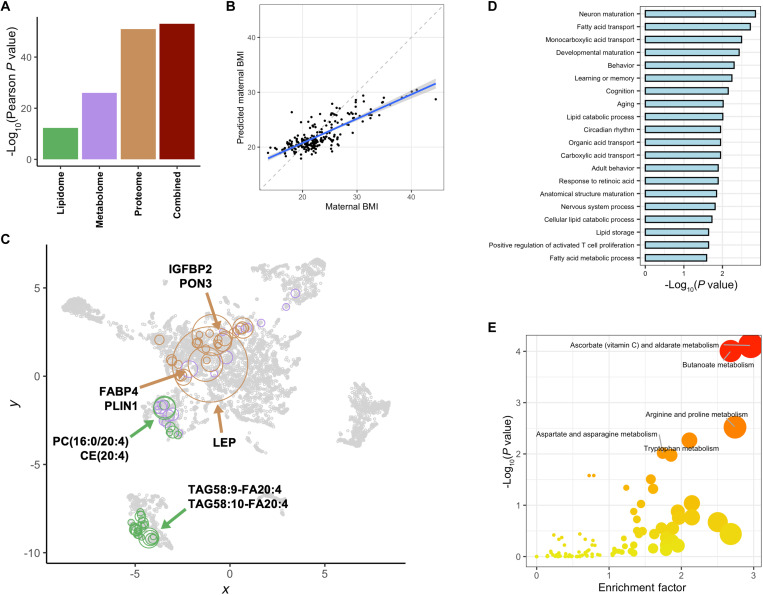
Predictive modeling of the maternal BMI. A cross-validated gradient-boosted tree (XGBoost) model for the prediction of maternal BMI was trained on each individual omic as well as the combined multiomic dataset. (**A**) Comparison of the cross-validated performance of each model for the prediction of maternal BMI. (**B**) Maternal BMI predictions of the combined multiomic model (Pearson’s *r* = 0.81, 95% CI: 0.76 to 0.85, *P* = 8.7 × 10^−54^, RMSE = 3.3, MAE = 2.5, *N* = 226). The blue line and blue shadow represent the regression line and 95% CI of the predicted values versus the ground truth. (**C**) Two-dimensional UMAP visualization of the multiomic features significantly correlated with maternal BMI. Each circle represents a feature, with sizes proportional to Spearman correlation with maternal BMI, and colors representing modality. (**D**) GO overrepresentation analysis performed on the plasma proteomic features significantly correlated with maternal BMI as assessed using Fisher’s exact test. (**E**) Metabolic pathway enrichment analysis performed on the metabolomic features significantly correlated with maternal BMI.

Maternal body mass index (BMI) is known to have a U-shaped relationship with PTB, with an increased risk of PTB at both ends of the BMI spectrum (*39*, *40*). To further understand the impact of BMI on maternal biology in the multiomics subcohort, a cross-validated XGBoost model was built with the integrated multiomic data, which was highly predictive of maternal BMI (*r* = 0.81, 95% CI: 0.76 to 0.85, *P* = 8.7 × 10^−54^, RMSE = 3.3, MAE = 2.5, *N* = 226) and revealed a particularly strong signature in the maternal proteome (*r* = 0.80, *P* = 1.1 × 10^−51^) (Fig. 6, A and B). Features significantly correlated with BMI consisted of proteins associated with metabolism and biomolecule storage and a cluster of highly intercorrelated lipid species (Fig. 6C). Adipocyte hormone leptin (LEP; Rho = 0.68), structural protein fatty acid binding protein 4 (FABP4; Rho = 0.51), and lipid-associated perilipin 1 (PLIN1; Rho = 0.41) positively correlated with maternal BMI, findings supported by previous reports (*41*–*43*). Conversely, insulin-like growth factor binding protein 2 (IGFBP2; Rho = −0.54) and high-density lipoprotein (HDL)–associated paraoxonase 3 (PON3; Rho = −0.40) exhibited inverse correlations with maternal BMI (*43*, *44*). The lipidome features most correlated with BMI all showed positive correlations and included lipids PC(16:0/20:4) (Rho = 0.43), CE(20:4) (Rho = 0.44), and TAG58:10-FA20:4 (Rho = 0.42).

GO term overrepresentation analysis performed on the proteome features significantly correlated with BMI indicated an enrichment of metabolic gene sets such as fatty acid transport (Fisher’s exact test *P* = 0.0019) and lipid catabolic process (Fisher’s exact test *P* = 0.0098) (Fig. 6D). Pathway analysis of the set of metabolites with a significant association with BMI revealed the enrichment of metabolism-related pathways like ​​ascorbate and aldarate metabolism (*P* = 7.7 × 10^−5^), butanoate metabolism (*P* = 9.9 × 10^−5^), and arginine and proline metabolism (*P* = 0.003) (Fig. 6E). A cross-validated XGBoost model built on the maternal proteome from the multiomics subcohort was further able to predict maternal BMI in an independent set of early pregnancy samples from the AMANHI and GAPPS biorepositories (Pearson’s *r* = 0.50, 95% CI: 0.29 to 0.67, *P* = 12.5 × 10^−5^, RMSE = 3.9, MAE = 2.9, *N* = 63) (fig. S12).

In summary, these results provide an integrated view of epidemiologically associated variables for PTB and their respective biological correlates. These observations highlight the unprecedented opportunity to study how such variables can affect maternal biology.

## DISCUSSION

This study leveraged a large, multinational cohort with harmonized data collection through two major biorepositories in LMICs to describe a generalizable epidemiological set of factors associated with PTB. We augmented those data with attendant high-dimensional proteomic, metabolomic, and lipidomic profiling of maternal plasma in a subcohort to build an integrated view of how such maternal factors, associated with PTB, can affect omics during pregnancy. The epidemiological model found both site-agnostic correlates and cohort-specific associations of PTB (fig. S2C), shedding light on the consistent risks of this complex disease as well as how other risks vary across populations. The integrated multiomic modeling of maternal factors revealed the biological signatures of the time from sampling to delivery, maternal age, gravidity, and BMI, deepening our understanding of maternal biological adaptations to pregnancy.

Predicting PTB using only information from early and mid pregnancy remains a challenge in clinical practice, with most screening procedures achieving modest performance (*45*, *46*). While complex machine learning models trained on electronic health record (EHR) data have achieved high performance, deploying these models in LMICs—where PTB is most prevalent—poses a challenge, limiting their scalability (*47*, *48*). The epidemiological model described here uses easily collectable data from the mother’s medical history and early and mid pregnancy antenatal care visits to build a generalizable model that works across four different LMICs with an overall AUROC of 0.70, a performance comparable to previously published predictive models of PTB (*49*–*52*). The model’s AUPRC of 0.27, while modest, reflects a Lift of 2.4 over the baseline prevalence of PTB in our cohort, which is in line with the Lift of 2.5 reported by the only comparable previous study that also used AUPRC for assessing model performance (*52*).

The epidemiological risk prediction of PTB inferred from our full cohort confirmed previously known associations between maternal covariates and incidence of PTB. The most useful features for the prediction of PTB spanned across all the clinical categories used, highlighting the importance of an integrated view of maternal health when predicting PTB. History of previous PTB, Cesarean delivery, stillbirth, or miscarriage were strongly associated with PTB in our cohort, all of which are known to be risk factors of PTB (*11*–*13*). Maternal anthropometry and overall health were also relevant for PTB prediction, with lower maternal height (*53*) and weight (*54*), diabetes (*55*), and hypertension (*56*) all contributing to increased risk of PTB. Notably, the epidemiological risk prediction included several social determinants of health, with a lowered incidence of PTB in women with higher levels of education (*57*, *58*) and active employment.

Determining the time-to-delivery using noninvasive methods (such as targeted measurements in blood and urine) is of high interest given their potential for scalability and deployment in LMICs (*28*). In our multiomic model, predictions for time-to-delivery were powered by a set of highly intercorrelated plasma proteins and metabolites, including multiple placental and fetal proteins and pregnancy-associated steroid hormones. Many of the proteins in this module have been previously associated with the chronicity of pregnancy (*23*, *27*, *34*, *59*), and the set of steroid hormones recapitulates what is known regarding the dynamics of estriol glucuronide and progesterone during the course of pregnancy (*24*, *60*), both of which validate our approach. We also observed a negative correlation between cortolone glucuronide and time-to-delivery (Rho = −0.40), contrasting with previous reports on cortisol-related hormone levels during pregnancy (*61*). This is possibly explained by a pregnancy-associated shift of the metabolism of cortisol-related metabolites, particularly since glucuronidation is a key modification for the excretion of steroid hormones (*62*). The minimal model for the prediction of time-to-delivery needed only three proteins to achieve the performance of the full model in an independent test set (Fig. 3F). Of these proteins, one was of fetal origin (AFP), while the other two were derived from the placenta (ALPP and SPINT1). Notably, low levels of SPINT1 have recently been shown to be a marker of placental insufficiency and fetal growth restriction (*63*, *64*). Together with our findings relating SPINT1 to the chronicity of pregnancy, these results highlight the need for further interrogation of the role of SPINT1 in the progression of pregnancy.

Recent work focusing on the dynamics preceding labor onset highlighted the importance of the interactions between plasma proteins and immune system cellular responses for maintaining fetal tolerance and initiating parturition (*25*). Mirroring this finding, we observed increasing levels of immune system–associated proteins such as PD-L1, CCL28, and LIFR in maternal plasma closer to the onset of labor. PD-L1 is a key regulator of T cell–mediated immunity and peripheral tolerance, and there is a growing body of evidence that it is also critical for the establishment and progression of a healthy pregnancy (*65*, *66*). CCL28 is a chemokine with diverse immune functions, including antimicrobial activity and immune regulation through the recruitment of regulatory T cells (T_regs_) to mucosal surfaces. LIFR and its ligand LIF play a role in maintaining a tolerogenic environment during pregnancy by promoting T_reg_ differentiation (*67*, *68*). Soluble PD-L1 levels have been described to increase in maternal plasma throughout pregnancy (*69*), while both CCL28 and LIFR levels were previously found to be elevated in the third trimester when compared to postpartum (*70*). Our results complement these findings by providing a longitudinal assessment of these immunological proteins during pregnancy, showing increasing levels of all three proteins, which could help maintain immune tolerance of the fetus, maintain an anti-inflammatory environment, and prevent an early onset of parturition.

Our work also revealed an age- and site-independent association between a woman’s gravidity and levels of eNOS and CXCL13 during early and mid pregnancy. This finding was further validated in an independent Stanford cohort, suggesting its generalizability across populations. Dysregulation of eNOS and CXCL13 has been previously associated with adverse pregnancy outcomes such as PTB (*71*, *72*) and preeclampsia (*73*). eNOS produces NO, a strong vasodilator, making eNOS critical for proper vascular and endothelial function and the regulation of blood pressure (*72*). CXCL13 is a potent lymphocyte—and particularly B cell—chemoattractant with antimicrobial and antiangiogenic activity that also plays a role in the recruitment of lymphocytes to sites of inflammation (*74*). Both eNOS and CXCL13 in the periphery have been observed to increase during pregnancy and originate from the placenta (*73*) and decidua (*75*), respectively. In these tissues, eNOS plays a key role in supporting maternal-fetal exchange through the production of NO (*76*), while CXCL13 is hypothesized to recruit B cells and T_regs_ to support maternal-fetal immune tolerance (*71*, *75*).

Our results suggest that multiparous women might have an incrementally decreased up-regulation eNOS and CXCL13, reflecting a form of “pregnancy memory” (*77*) in which maternal adaptations to pregnancy affect future pregnancies. Pregnancy memory has previously been studied in the context of the prediction of PTB (*59*), specifically given that having a previous PTB and nulliparity are both risk factors of PTB (*8*, *9*, *78*). In particular, permanent epigenetic changes (*79*) and the development of immunological memory to fetal antigens (*80*) after the first pregnancy guides the healthy progression of subsequent pregnancies. On the basis of our findings, previous experience of a successful embryonic implantation and establishment of a functional maternal-fetal interface could lead to a decreased need to up-regulate eNOS and CXCL13 in the beginning of subsequent pregnancies. While our multiomics subcohort allowed us to show this in early and mid pregnancy, the Stanford validation cohort further illustrated the differences seen regarding CXCL13 levels between nulliparous and parous women across pregnancy and postpartum (fig. S11). In the Stanford cohort, each trimester showed increased levels of CXCL13 in the nulliparous women, with the difference between groups decreasing with the progression of pregnancy and vanishing postpartum. However, it has also been proposed that pregnancies permanently impair the responsiveness of the maternal vasculature to NO, leading to oxidative stress, reduced eNOS expression, and endothelial dysfunction in multiparous women (*81*), which could also explain our findings but does not address why nulliparity is a risk factor for adverse pregnancy outcomes. To more completely understand how a mother’s obstetric history will affect her risk of complications in future pregnancies, further studies on how maternal adaptations to pregnancy persist as pregnancy memory are needed.

Both low and high maternal BMI have been shown to increase the risk of PTB, making the impact of BMI on prematurity difficult to disentangle (*39*, *40*). In the full cohort, maternal BMI had a weak positive correlation with GA at delivery (Rho = 0.08, *P* = 6.4 × 10^−22^, *N* = 13,200) and a moderate association with birth weight (Rho = 0.29, *P* = 1.8 × 10^−213^, *N* = 11,985), echoing previously reported findings (*82*). After stratifying by low (<18.5 kg/m^2^) and high (>25.0 kg/m^2^) BMI, we saw an increased risk of PTB in underweight women [risk ratio (RR) = 1.33, 95% CI = 1.16 to 1.51, *P* = 3.0 × 10^−5^] when compared to women in the reference range, but no substantial increased risk was observed in women with high BMI when compared with those with normal BMI (RR = 1.07, 95% CI = 0.95 to 1.19, *P* = 0.26). These results are possibly due to the nature of the populations included in the study, particularly the study setting in LMICs. Maternal BMI had a significant impact on the maternal proteome, metabolome, and lipidome, with our cross-validated multiomic model exhibiting strong performance in predicting BMI (*r* = 0.80). Model predictions were driven by plasma proteins previously associated with BMI, such as LEP, FABP4, and IGFBP2 (*41*, *42*). Notably, dysregulation of these proteins has also been associated with pregnancy complications such as gestational diabetes mellitus (*83*) and PTB (*84*, *85*). Our work therefore confirms the relationship between maternal BMI and specific plasma proteins with previously described roles in adverse pregnancy outcomes.

This study had certain limitations. First, a case-control study design was used when selecting participants for the multiomics subcohort so that the findings described here were not inferred from a random subset of the full cohort. While the epidemiological cohort was clinically highly heterogeneous, this heterogeneity was not fully reflected in the multiomics subcohort due to the design used to select participants for multiomic profiling. The homogeneity of the multiomics subcohort thus limited the degree of inference that could be applied using the multiomic data to understand the biological impact of the variables most associated with PTB. Second, the sample size, while unprecedented for this type of multiomic study, is small compared to the substantially larger dimensionality of the feature space, which leads to problems with overfitting and lack of generalizability. However, the significant associations and models presented in this study were validated where possible with independent data, demonstrating the robustness of some of our observations and predictive models. Third, the clinical data collected lacked fasting status and nutritional status, both of which are critical to contextualize the findings presented in this study. Specifically, recent diet and nutritional quality can affect the abundance of metabolites and proteins and the risk of PTB, which could inform some of the results shown. Fourth, our study lacks systemic profiling of the immune system by technologies such as mass cytometry, which will be critical to gain further insight into the potential biological mechanisms proposed here. Overall, future studies with larger omic sample sizes and a more clinically diverse population will be needed to validate the findings described here, particularly as it relates to fully integrating the epidemiological variables with the multiomic data to predict PTB.

Our study provides an integrated view of how previously identified epidemiological associations such as parity, BMI, and age are etiologically connected to an underlying biology evidenced by various omic correlates. This integrated view was possible by leveraging a large, multinational cohort together with multiomic profiling in a subcohort to simultaneously explore the population-level correlates of PTB and how these associate with individual women’s biology. Furthermore, our results put forward a conceptual framework for studying the biological mechanisms underlying some of the long-standing observed population-level risk factors of PTB. In addition, more frequent prenatal visits are associated with lower PTB rates in LMICs (*16*), suggesting that early identification of at-risk pregnancies could enable targeted use of more frequent antenatal visits, a limited resource in LMICs. Finally, this approach promises to address the lack of generalizability that plagues predictive models for PTB and provides insights for the development of biological and socioeconomic interventions to prevent PTB that can be generalized across multiple populations.

## MATERIALS AND METHODS

### Experimental design

The study population comprised pregnant women selected from five biorepository-supported cohorts in Matlab, Bangladesh; Lusaka, Zambia; Sylhet, Bangladesh; Karachi, Pakistan; and Pemba, Tanzania. The study was approved by the Stanford University Institutional Review Board, and ethical exemptions were sought and obtained independently from the respective country by each birth cohort supported by the AMANHI and GAPPS biorepositories. Written informed participant consent was obtained from each participant in the original cohorts and extends to the present study. No compensation or incentives were provided for participating in this study. We followed the Transparent Reporting of a Multivariable Prediction Model for Individual Prognosis or Diagnosis (TRIPOD) reporting guideline. This study analyzed plasma collected from May 2014 to August 2018, and data were analyzed from October 2020 to August 2021.

GA at the time of sampling was determined by clinical and ultrasonographic assessment. To focus on prediction of PTB with measurements taken before 28 weeks GA, pregnant women enrolled in the study with an extremely preterm birth (GA at birth less than 28 weeks) were excluded from the analysis, as were pregnant women with only one antenatal care visit before 28 weeks.

The multiomics subcohort was chosen in a case-control design and included 113 pregnant women with a PTB (48.9%) and 118 with a term delivery (51.1%). Samples were collected from singleton pregnancies without congenital malformations and with spontaneous live deliveries. Case and control samples were matched within each site based on GA at sampling, and maternal covariates were not significantly different between cases and controls. Missing values were imputed using mean imputation.

### Biological assays

At all AMANHI and GAPPS sites, trained phlebotomists or nursing staff collected blood samples. After centrifugation, plasma was aliquoted and stored at −80°C until future analyses. Collection and processing of plasma samples were performed according to harmonized operating procedures at all study cohorts. Blood collected in EDTA tubes was cold centrifuged at 3000 rpm for 10 min within 4 hours of collection. Plasma was separated and stored at −80°C until shipment. From each repository, 0.5 ml of plasma for proteome, 0.5 ml of plasma for metabolome, and 0.5 ml of plasma for lipidome analysis were shipped. Samples were shipped on dry ice as a single batch and under continuous temperature monitoring.

Lipidomic and metabolomic data were generated using targeted mass spectrometry and untargeted liquid chromatography–mass spectrometry (LC-MS). Metabolites and complex lipids were extracted using a biphasic separation with cold methyl tert-butyl ether (MTBE), methanol, and water in a deep-well plate format. Metabolite extracts were analyzed using a broad-spectrum untargeted LC-MS platform as previously described (*86*), while complex lipids were quantified using a targeted MS-based approach (*87*). Metabolite data from each mode were independently analyzed using Progenesis QI software (v2.3) (Nonlinear Dynamics, Durham, NC), and metabolite abundances were reported as spectral counts. Lipidyzer data were reported by the Lipidomics Workflow Manager (LWM; v1.0.5.0) software, which calculates concentrations for each detected lipid as average intensity of the analyte multiple reaction monitoring (MRM)/average intensity of the most structurally similar internal standard (IS) MRM multiplied by its concentration. Lipid abundances were reported as concentrations in nmol/g.

The proteomic analysis was performed by O-link Proteomics (Watertown, MA) with a highly multiplex proteomic platform using proximity extension technology, which quantified relative amounts of protein via real-time polymerase chain reaction (*88*). For this study, 13 panels were used, each measuring 92 different proteins simultaneously in 1 μl of plasma. Relative amounts of protein were quantified as normalized protein expression (NPX). NPX was derived by subtracting the Ct value of the extension control reaction from the raw Ct value (threshold cycle) to adjust for technical variations (dCt), then subtracting differences in Ct values between plates (inter-plate control) from the dCt value (ddCt value) to adjust for inter-assay variability, and then subtracting the ddCt value from a correction factor to adjust for background noise and invert the scale. See the Supplementary Materials for more details.

### Statistical analyses

All analyses were performed with R version 4.0.4. A repeated cross-validation scheme was used in all multivariate modeling to prevent overfitting and get an estimate of the performance of the model on unseen participants. In brief, participants were randomly split evenly into a training set (50%) and a test set (50%). A gradient-boosted tree (XGBoost) (*35*) model was built on the training data (see the Supplementary Materials for hyperparameters used). The XGBoost model was then used to predict the outcome for the participants in the test data. This procedure was performed 50 times using different train and test sets in each iteration, and final test predictions for each participant were generated by averaging the participant’s predictions from the iterations in which the participant was present in the test set. For the prediction of PTB in the full cohort, a weighted loss was used to address the class imbalance present in the data (Table 1). Model performance was assessed using AUROC and AUPRC for classification tasks. For regression tasks, the Pearson correlation coefficient (Pearson’s *r*) between model predictions and ground truth was used.

### Feature selection for minimal model

The feature selection paradigm consisted of repeated subsampling of the training set to build an XGBoost model in the sampled participants using the full feature set. In each iteration, the top 10 features were picked based on feature importance. After 100 iterations, the 3 features picked with the highest frequency were used to train a cross-validated minimal model on the whole training set and to make predictions on the independent test set. Model performance was then assessed in the cross-validated training set and the independent test set using Pearson’s *r*.

### Multiomic interactome analysis

The multiomic interactome was described by calculating the Spearman correlation coefficients within (intra-) and between (inter-) omics. The Bonferroni correction was applied to control for multiple hypothesis testing. A Bonferroni-adjusted *P*-value significance threshold of 0.05 was used, which corresponded to an absolute Spearman correlation coefficient threshold of 0.38 based on the *t* distribution with 229 degrees of freedom. To generate a background distribution of random correlations, a set of random features was generated by sampling each feature with replacement and performing the same correlation calculation on the random feature set. Interomic correlations were further visualized using a chord diagram generated in R with the circlize package (version 0.4.13) (*89*). The number of significant interomic correlations was normalized to the total number of possible correlations between each omic pair to visualize the relative contribution of each omic pair to the interactome.

### Correlation network generation

The maternal and fetal covariates collected during the study were visualized using an MST where nodes represent covariates and are grouped by clinical categories. The underlying correlation structure for each clinical category was calculated using the Spearman correlation matrix and then reduced using an MST. The full MST was extracted by integrating the MSTs for each clinical category together and then arranged into a two-dimensional layout using the Fruchterman-Reingold force-directed algorithm (*90*). Edges were drawn between significantly associated covariates after adjusting for multiple hypothesis testing using Bonferroni’s correction. The color and size of each node are proportional to the −log_10_(Wilcoxon rank sum test *P* value) of the covariate’s association with PTB and the −log_10_(Pearson *P* value) of the covariate’s predictability using multiomic data, respectively. The thickness of each edge is proportional to the Spearman’s rank correlation coefficient between the two covariates.

### Pathway enrichment analysis

Pathway enrichment was performed on the top proteomic and metabolomic features associated with the clinical covariates of interest. Top proteomic features were analyzed using gene set overrepresentation analysis of GO terms (*32*) performed in R with the topGO package (version 2.42.0) (*31*). Specifically, Fisher’s exact test was used to determine the enrichment of each GO term in the Biological Process ontology. Top metabolomic features were analyzed in R using the Mummichog algorithm of the MetaboAnalystR package (version 3.0.3) (*33*). The metabolic model used was *Homo sapiens* MFN, and significance was assessed by Fisher’s exact test.

### Validation cohorts

Previous studies of pregnancy with comparable multiomic approaches were used for independent validation of hypotheses inferred from observations made in this study. Particularly, these studies used similar multiomic modalities and had publicly available data.

AMANHI and GAPPS pilot study (*27*): This study profiled 81 pregnant women from five AMANHI and GAPPS biorepository-supported sites. Plasma and urine samples were collected during early pregnancy and used to generate proteomic, metabolomic, and lipidomic datasets.

Lucile Packard Children’s Hospital at Stanford University study (*37*): This study profiled 17 pregnant women from Lucile Packard Children’s Hospital. Peripheral blood, plasma, serum, and culture swab samples were collected at each trimester and postpartum and used to generate immunome, transcriptome, microbiome, proteome, and metabolome datasets.

### *P*-value adjustment

All *P* values were adjusted for multiple hypothesis testing using the Bonferroni correction where appropriate, in which each raw *P* value is multiplied by the number of statistical tests performed to calculate the adjusted *P* value.

## Supplementary Materials

### This PDF file includes:

Supplementary Methods Figs. S1 to S12 Legends for data S1 and S2 References

## Notes

View/request a protocol for this paper from *Bio-protocol*.
