# Supplementary material for: Multiomic signals associated with maternal epidemiological factors contributing to preterm birth in low- and middle-income countries
Source: Sci Adv. 2023 May 26;9(21):eade7692. doi: 10.1126/sciadv.ade7692 (PMC10208584; doi:10.1126/sciadv.ade7692)
Supplement: Supplementary file 1 — Supplementary Methods Figs. S1 to S12 Legends for data S1 and S2 References [file sciadv.ade7692_sm.pdf]

Science Advances
AAAS

# Supplementary Materials for

## Multiomic signals associated with maternal epidemiological factors contributing to preterm birth in low- and middle-income countries

Camilo A. Espinosa *et al.*

Corresponding author: Nima Aghaeepour, naghaeep@stanford.edu

*Sci. Adv.* **9**, eade7692 (2023)
DOI: 10.1126/sciadv.ade7692

**The PDF file includes:**

Supplementary Methods
Figs. S1 to S12
Legends for data S1 and S2
References

**Other Supplementary Material for this manuscript includes the following:**

Data S1 and S2

## Supplementary Methods

*Study design*

Our study population consisted of 231 pregnant women selected from the following GAPPS- and AMANHI-supported birth cohorts: (1) the GAPPS Preterm and Stillbirth Study in Matlab, Bangladesh (PreSSMat Study, icddr,b, Matlab, Bangladesh), a prospective cohort study designed to assess biological, environmental, and social determinants of adverse pregnancy outcomes; (2) the GAPPS Preventing Preterm Birth Initiative in Zambia (ZAPPS Study, UNC-CH/UTH, Lusaka, Zambia) (91), a prospective cohort study and biorepository designed to characterize the factors associated with PTB and outcomes in Zambia; and (3) the Alliance for Maternal and Neonatal Health Improvement (AMANHI) biorepository study in Bangladesh Sylhet, Pakistan Karachi and Pemba Tanzania. All pregnant women provided written informed consent for participation in the original study, and for future utilization of specimens. For the current studies ethical exemptions were sought from the respective country IRBs and regulated under necessary material transfer and data transfer agreements.

At all AMANHI and GAPPS cohorts, trained phlebotomists collected blood samples for centrifugation and aliquoting of serum, plasma, and buffy coat for storage and future analyses. In addition, maternal urine was collected in parallel. With a view to facilitate the future of omics study, special care was taken to ensure sample storage at -80°C in each biobank. Unique study identification numbers were assigned to all samples, which were linked to each participant. Outcome assessment was done by birth surveillance through phone calls and household visits (92).

Collection and processing of all sample types was performed following standard operating procedures at all study cohorts (93). Blood collected in EDTA tubes was cold centrifuged at 3,000 rpm for 10 mins within 4 hrs. Plasma was separated and stored at -80°C until shipment. From each repository, 0.5 mL of plasma for proteome, 0.5 mL of plasma for metabolome, and 0.5 mL of plasma for lipidome analysis were shipped. Samples were shipped on dry ice as a single batch and under continuous temperature monitoring.

*Clinical covariates*

Field workers were trained to collect detailed phenotypic and demographic data from the women and their families through scheduled household visits during pregnancy and post-partum. Clinical covariates were manually harmonized across all five cohorts.

*Metabolomics and lipidomics*

*Sample preparation:* Metabolites and complex lipids were extracted using a biphasic separation with cold methyl tert-butyl ether (MTBE), methanol, and water in deep well plate format.

Briefly, 1 ml of ice-cold MTBE and 260 µl methanol was added to 40 µl of the plasma spiked-in with 40 µl deuterated lipid internal standards (Sciex, cat# 5040156, lot# LPISTDKIT-103). The samples were then agitated at 4°C for 30 min. After addition of 250 µl of ice-cold water, the samples were vortexed for 1 min and centrifuged at 3,800 g for 5 min at 4°C. The upper organic phase contains the lipids, the lower aqueous phase contains the metabolites, and the proteins are precipitated at the bottom of the well. For quality control, 3 reference plasma samples (40 µl plasma) as well as one control sample lacking any sample were processed in parallel per plate.

1. **Metabolites*:*** Proteins were further precipitated by adding 500 µl of 1:1:1 acetone:acetonitrile:methanol spiked-in with 15 labeled metabolite internal standards to 300 µl of the aqueous phase and 200 µl of the lipid phase and incubating the samples overnight at -20°C. After centrifugation at 3,800 g for 10 min at 4°C, the metabolic extracts were dried down to completion and resuspended in 200 µl 50/50 methanol/water for LC-MS analysis.

2. **Complex lipids:** 700 µl of the organic phase was dried down under a stream of nitrogen and resolubilized in 200 µl of methanol for storage at -20°C until analysis. The day of the analysis, samples were dried down, resuspended in 300 µl of 10 mM ammonium acetate in 90:10 methanol:toluene and centrifuged at 3,800 g for 5 min at 4°C.

*Data acquisition:* Metabolite extracts were analyzed using a broad-spectrum untargeted LC-MS platform as previously described (86) while complex lipids were quantified using a targeted MS-based approach (87).

1. **Untargeted Metabolomics by Liquid Chromatography (LC)-MS:** Metabolic extracts were analyzed four times using HILIC and RPLC separation in both positive and negative ionization modes. Data were acquired on a Thermo Q Exactive HF mass spectrometer for HILIC (Thermo Fisher Scientific, Bremen, Germany) and a Thermo Q Exactive mass spectrometer for RPLC (Thermo Fisher Scientific, Bremen, Germany). Both instruments were equipped with a HESI-II probe and operated in full MS scan mode. MS/MS data were acquired on quality control samples (QC) consisting of an equimolar mixture of all samples in the study. HILIC experiments were performed using a ZIC-HILIC column 2.1 x 100 mm, 3.5 µm, 200Å (Merck Millipore, Darmstadt, Germany) and mobile phase solvents consisting of 10 mM ammonium acetate in 50/50 acetonitrile/water (A) and 10 mM ammonium acetate in 95/5 acetonitrile/water (B). RPLC experiments were performed using a Zorbax SBaq column 2.1 x 50 mm, 1.7 µm, 100Å (Agilent Technologies, Palo Alto, CA) and mobile phase solvents consisting of 0.06% acetic acid in water (A) and 0.06% acetic acid in methanol (B). Data quality was ensured by (i) injecting 6 and 12 pool samples to equilibrate the LC-MS system prior to running the sequence for RPLC and HILIC, respectively, (ii) injecting a pool sample every 10 injections to control for signal deviation with time, and (iii) checking mass accuracy, retention time and peak shape of internal standards in each sample.

2. **Targeted Lipidomics using the Lipidyzer Platform:** Lipid extracts were analyzed using the Lipidyzer platform that comprises a 5500 QTRAP system equipped with a SelexION differential mobility spectrometry (DMS) interface (Sciex) and a high flow

LC-30AD solvent delivery unit (Shimazdu, Columbia, MD). Briefly, lipid molecular species were identified and quantified using multiple reaction monitoring (MRM) and positive/negative ionization switching. Two acquisition methods were employed covering 13 lipid classes; method 1 had SelexION voltages turned on while method 2 had SelexION voltages turned off. Data quality was ensured by i) tuning the DMS compensation voltages using a set of lipid standards (cat# 5040141, Sciex) after each cleaning, more than 24 hours of idling or 3 days of consecutive use, ii) performing a quick system suitability test (QSST) (cat# 5040407, Sciex) before each batch to ensure acceptable limit of detection for each lipid class, and iii) triplicate injection of lipids extracted from a reference plasma sample (cat# 4386703, Sciex) at the beginning of the batch.

*Data processing*

1. **Metabolomics:** Data from each mode were independently analyzed using Progenesis QI software (v2.3) (Nonlinear Dynamics, Durham, NC). Metabolic features from blanks and those that didn't show sufficient linearity upon dilution in QC samples ($r<0.6$) were discarded. Only metabolic features present in >2/3 of the samples were kept for further analysis. Missing values were imputed by drawing from a random distribution of low values in the corresponding sample. Intensity drift was corrected using the SERRF (systematic error removal using random forest) method (94). Data from each mode were merged and metabolic features were annotated as follows. Peak annotation was first performed by matching experimental m/z, retention time, and MS/MS spectra to an in-house library of analytical-grade standards. Remaining peaks were identified by matching

experimental m/z and fragmentation spectra to publicly available databases including HMDB, MoNA, MassBank, METLIN, and NIST using the R package ‘metID’ (v0.2.0). We used the Metabolomics Standards Initiative (MSI) level of confidence to grade metabolite annotation confidence (level 1 - level 3). Level 1 represents formal identifications where the biological signal matches accurate mass, retention time, and fragmentation spectra of an authentic standard run on the same platform. For level 2 identification, the biological signal matches accurate mass and fragmentation spectra available in one of the public databases listed above. Level 3 represents putative identifications that are the most likely name based on previous knowledge. Metabolite abundances were reported as spectral counts.

2. **Targeted Lipidomics:** Lipidyzer data were reported by the Lipidomics Workflow Manager (LWM, v1.0.5.0) software which calculates concentrations for each detected lipid as average intensity of the analyte MRM/average intensity of the most structurally similar internal standard (IS) MRM multiplied by its concentration. Lipids detected in less than 2/3 of the samples were discarded and missing values were imputed by drawing from a random distribution of low values class-wise in the corresponding sample. Lipid abundances were reported as concentrations in nmol/g.

<u>Proteomics</u>

The proteomic analysis was performed by O-link Proteomics (Watertown, MA) with a highly multiplex proteomic platform using proximity extension technology (88). For this study, thirteen panels were used, each measuring 92 different proteins simultaneously in 1μL of plasma. Each

protein was detected by a matched pair of antibodies that were coupled to unique and partially complementary oligonucleotides. When in close proximity, a new and protein-specific DNA reporter sequence was formed by hybridization and extension, which was then amplified and quantified by real-time PCR.

Relative amounts of protein were quantified as normalized protein expression (NPX). NPX was derived by subtracting the Ct value of the extension control reaction from the raw Ct-value (threshold cycle) to adjust for technical variations (dCT), then subtracting differences in Ct-values between plates (inter-plate control) from the dCt-value (ddCt-value) to adjust for inter-assay variability, and then subtracting the ddCt-value from a correction factor to adjust for background noise and invert the scale. An increase of 1 NPX corresponded to a doubling of the relative protein concentration (log 2 scale).

Quality control (QC) was performed at the assay and sample level. At the assay level (internal controls) each sample was spiked with two non-human antigens (incubation control), an antibody coupled with a unique pair of DNA tags (extension control), and a double-stranded DNA amplicon (detection control) to monitor the three major procedural steps (immunoreaction, extension, and amplification/detection). At the sample level three controls were added to each plate. A synthetic sample containing 92 antibodies with one pair of unique DNA tags in fixed proximity was added in triplicate to monitor and compensate for inter-run and inter-plate variations (inter-plate control). A negative control was added in triplicate to monitor for background noise. Finally, a pooled plasma sample was added in duplicate to monitor for intra- and interassay variability and determine coefficient of variations. A plate passes QC if the

standard deviation of internal controls was less than 0.2 NPX. Individual samples pass QC if values of internal controls deviated by less than 0.3 NPX from the plate median. In this study, all plates passed quality control, as did 98.2% of the plasma samples. Of all assayed proteins, 84.4% were detected in more than 75% of samples. The median intra-assay coefficient of variation was 6%. Prior studies have demonstrated strong associations between this assay and ELISA analysis (e.g., (95–97)).

*Model hyperparameters*

Hyperparameters for the gradient-boosted tree (XGBoost) models were as follows:

- *Models for Classification:* objective = “binary:logistic”; eval_metric = “aucpr”, nrounds = 15; scale_pos_weight = 1.4.
- *Models for Regression:* objective = “reg:squarederror”; eval_metric = “rmse”, nrounds = 10.

## Supplementary Figures

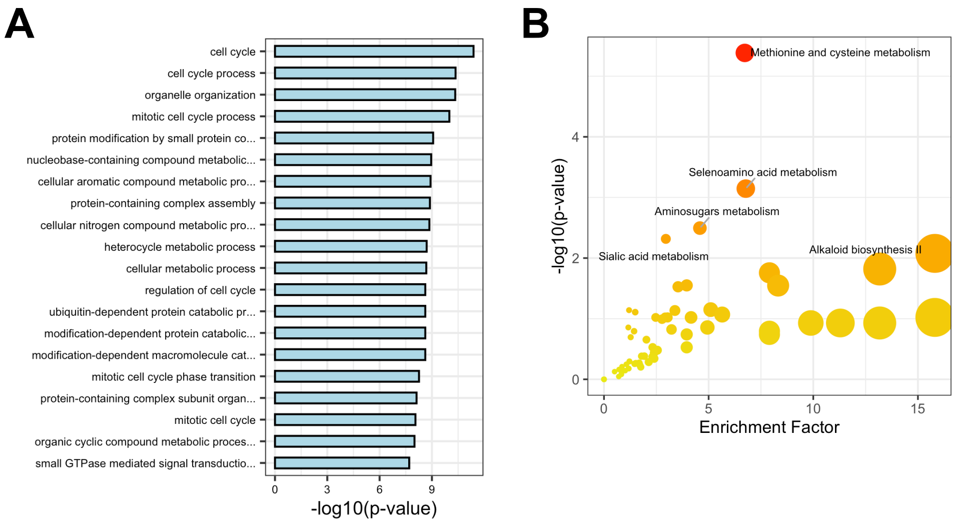


**Figure S1. Pathway analysis of strongly-correlated proteomic and metabolomic features.** Plasma samples taken during early and mid pregnancy from a subcohort of 231 women were analyzed to generate targeted lipidomic, untargeted metabolomic, and targeted proteomic datasets. (**A**) Gene ontology (GO) overrepresentation analysis performed on the plasma proteomic features with strong correlations (|Spearman's Rho| > 0.8) with metabolomic features, where the significance of each GO term is assessed using Fisher's exact test. (**B**) Metabolic pathway enrichment analysis performed on the metabolomic features with strong correlations (|Spearman's Rho| > 0.8) with plasma proteomic features, with the top 5 pathways annotated by significance.

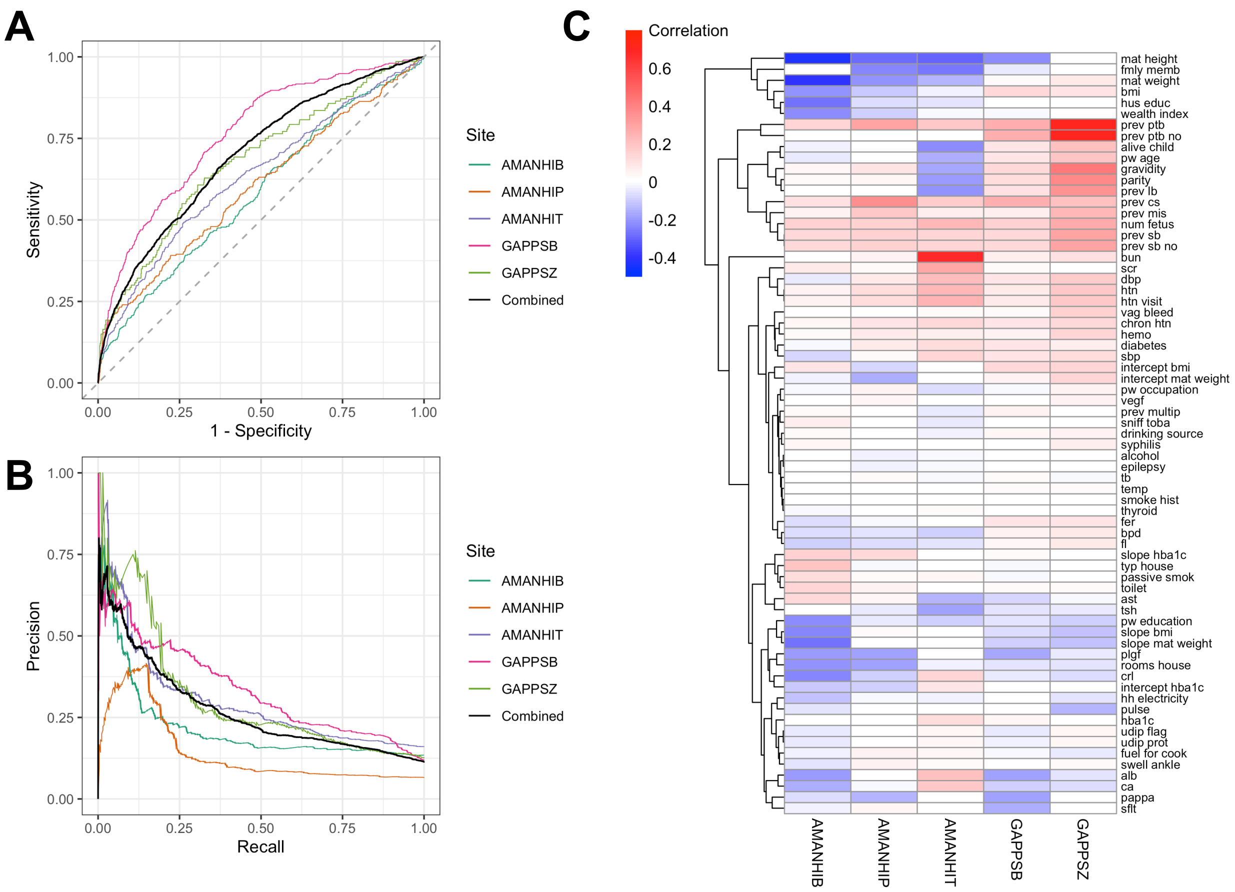


**Figure S2. Site-specific analysis of the epidemiological model of preterm birth (PTB).** A cross-validated gradient-boosted tree (XGBoost) model for the prediction of PTB was trained on the epidemiological data of the full cohort of 13,841 pregnant women. (**A**) Receiver-operating characteristic (ROC) curve for the epidemiological model stratified by site. (**B**) Precision-recall curve (PRC) for the epidemiological model stratified by site. (**C**) Heatmap of the Spearman correlation between the epidemiological model preterm risk score and each input feature stratified by site. See **Supplementary Materials** for more details on the clinical covariates.

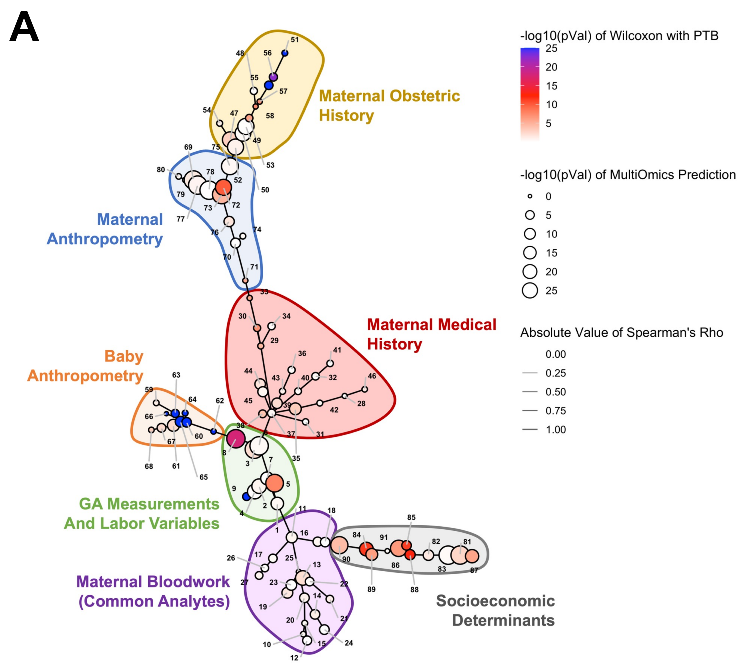


**Figure S3. Correlation network of the maternal and fetal covariates collected during the study.** Spearman correlations between all covariates were obtained and used to create a correlation network which was arranged into a 2-dimensional layout using a minimum spanning tree. Each node represents a different covariate, where the color of the node represents the strength of the univariate association of the covariate with preterm birth (PTB) and the size of the node represents the predictability of the covariate using multiomic data form the multiomics subcohort. Nodes are grouped by clinical categories and labeled with Covariate IDs, as listed in the **Supplementary Materials**.

A

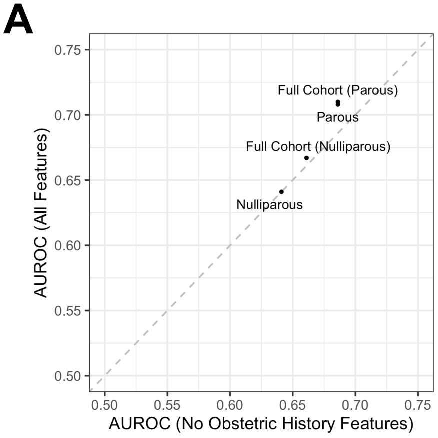


**Figure S4. Characterizing the role of obstetric history on the epidemiological model for preterm birth (PTB).** Cross-validated gradient-boosted tree (XGBoost) models for the prediction of PTB were built with and without obstetric history features in the nulliparous, parous, and combined populations. Their performance was assessed and compared using the area under the receiver operating characteristic curve (AUROC).

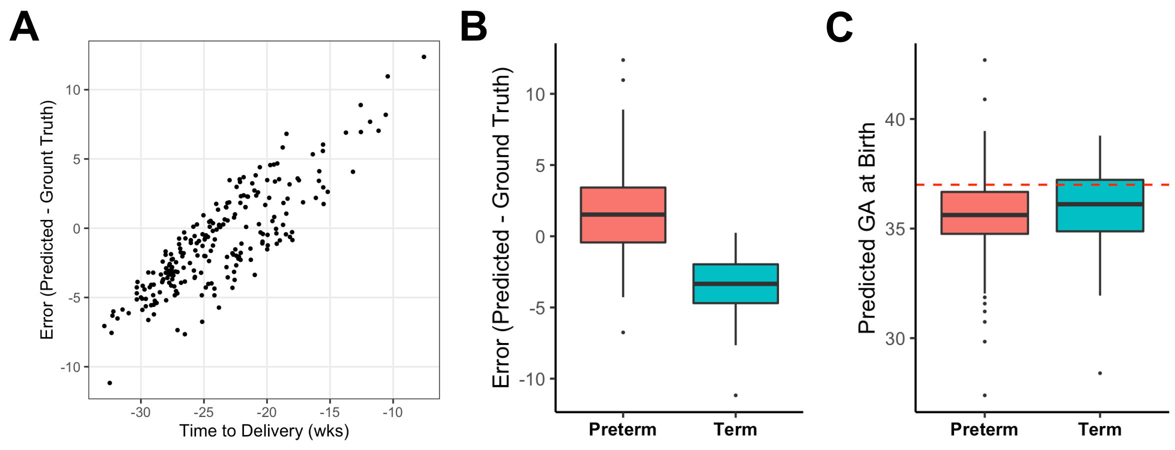


**Figure S5. Error analysis of the multiomic model of time-to-delivery.** A cross-validated gradient-boosted tree (XGBoost) model for the prediction of time from sampling to delivery was trained on the combined multiomic dataset with data from all sites. (**A**) Model error, defined as the difference between the predicted time-to-delivery and the actual time-to-delivery. (**B**) Model error stratified by preterm birth (PTB) status. (**C**) Predicted gestational age (GA) at birth as determined by the time-to-delivery model stratified by PTB status. The dashed red line at 37 weeks of GA indicates the boundary between a PTB and a term birth.

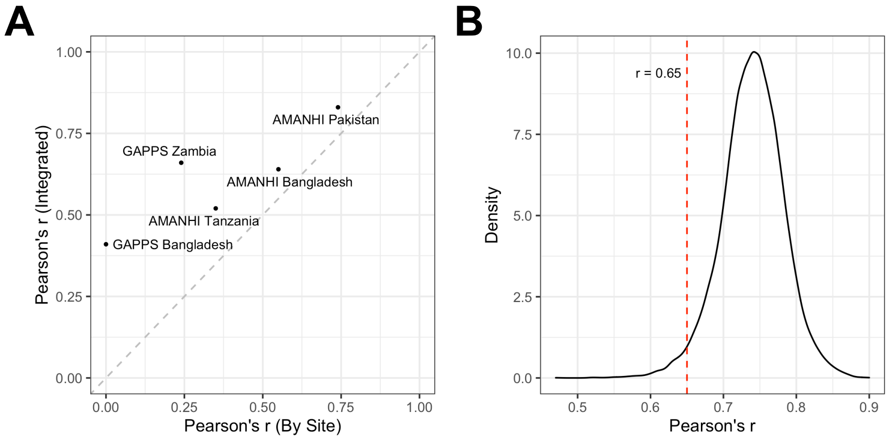


**Figure S6. Generalizability of the multiomic model of time-to-delivery across populations and preterm birth (PTB) rates.** A cross-validated gradient-boosted tree (XGBoost) model for the prediction of time from sampling to delivery was trained on the combined multiomic dataset with data from all sites. (**A**) Site-specific cross-validated XGBoost models for the prediction of time-to-delivery were trained and their site-specific performance was compared to the site-specific performance of the integrated model. (**B**) Random cohorts with PTB rates of 11.4% were subsampled from the multiomics subcohort and the performance of the model for the prediction of time-to-sampling to delivery in the sampled cohorts was assessed. Subsampled cohorts had at least 50 participants, and 10000 random cohorts were sampled.

A

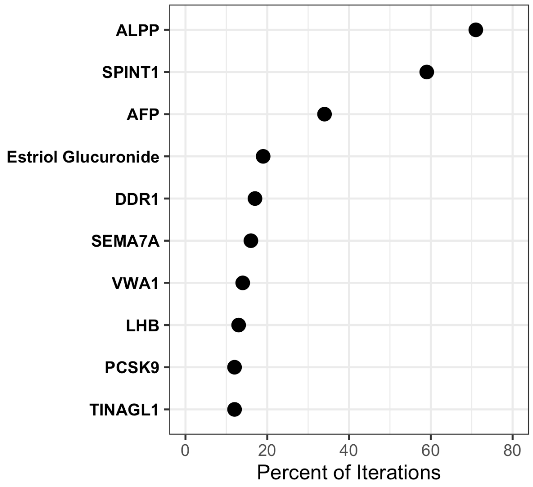


**Figure S7. Feature selection frequency in a reduced model for predicting time-to-delivery.** Participants were randomly split into a training set (*N* = 164, 70%) and a test set (*N* = 67, 30%). A repeated subsampling approach on the training set was used to select 3 features to build a minimal XGBoost model for the prediction of time-to-delivery. Plot depicts top features by the percent of the subsampling iterations the feature was picked in the feature selection paradigm. ALPP: Alkaline Phosphatase, Placental; SPINT1: Serine Peptidase Inhibitor, Kunitz Type 1; AFP: Alpha Fetoprotein; TINAGL1: Tubulointerstitial Nephritis Antigen Like 1; LHB: Luteinizing Hormone Subunit Beta; VWA1: Von Willebrand Factor A Domain Containing 1; IGSF3: Immunoglobulin Superfamily Member 3. DDR1: Discoidin Domain Receptor Tyrosine Kinase 1.

A

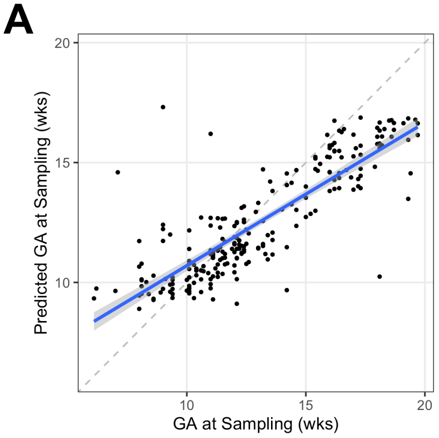


**Figure S8. A multiomic clock of gestational age (GA).** A cross-validated gradient-boosted tree (XGBoost) model for the prediction of GA at sampling was trained on the combined multiomic dataset. Scatterplot depicts GA predictions of the multiomic model (Pearson's $r = 0.84$, 95% confidence interval (CI): 0.80-0.87, $P = 1.4*10^{-62}$, RMSE = 1.9 weeks, MAE = 1.4 weeks, $N = 231$). The blue line and blue shadow represent the regression line and 95% CI of the predicted values versus the ground truth.

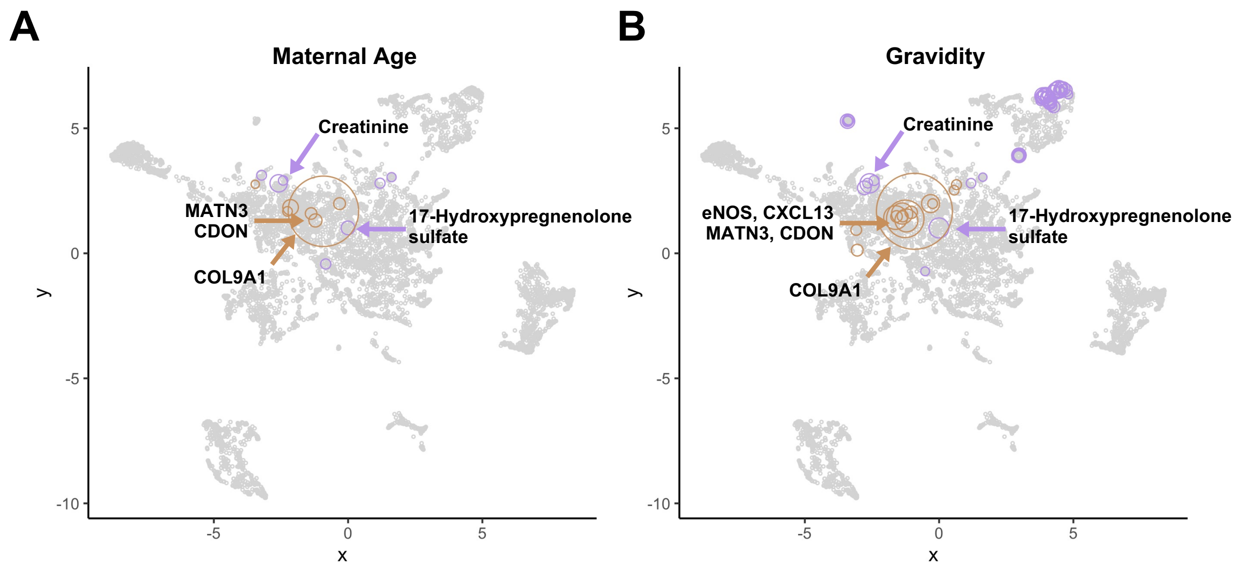


**Figure S9. Multiomic correlates of maternal age and gravidity.** Two-dimensional UMAP visualization of the multiomic features significantly correlated with maternal age (**A**) and gravidity (**B**), where each circle represents a feature, the size of the circle is proportional to the strength of the Spearman correlation with the relevant covariate, and the color of the circle represents the modality from which the feature originates.

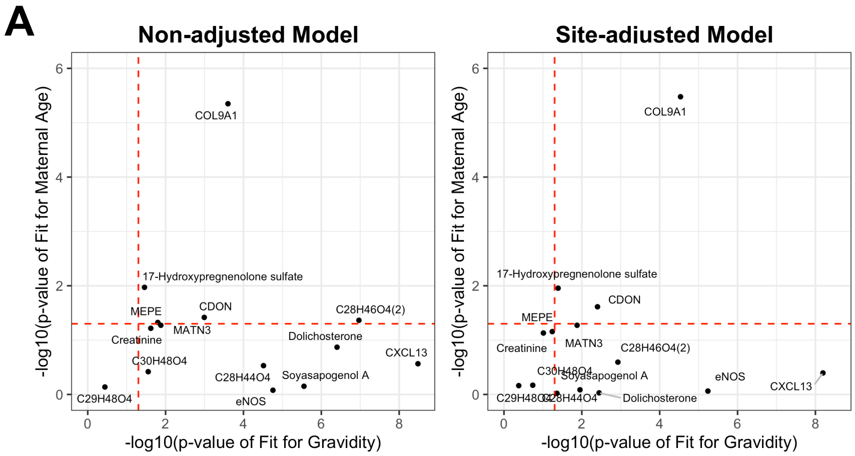


**Figure S10. Site-adjusted models of features significantly associated with maternal age and gravidity.** Significance of the fit of a multiple linear regression for each feature against maternal age and gravidity. Left: Multiple regressions performed without adjusting for site-specific effects. Right: Multiple regressions performed adjusting for site-specific effects using a mixed-effects model using random intercepts for each site.

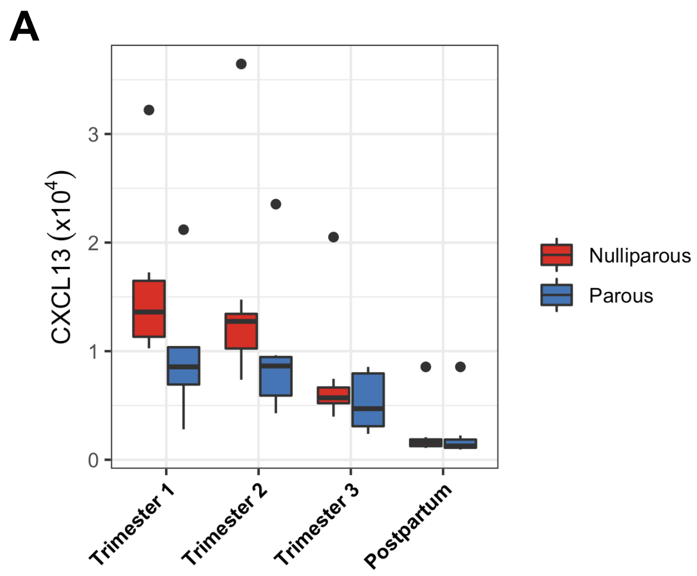


**Figure S11. CXCL13 levels in plasma differentially vary throughout pregnancy in nulliparous women.** The plasma proteome was measured at each trimester and postpartum in a cohort from Lucile Packard Children's Hospital at Stanford (*N* = 17). Boxplot depicts CXCL13 levels in plasma in each trimester and postpartum stratified by nulliparity.

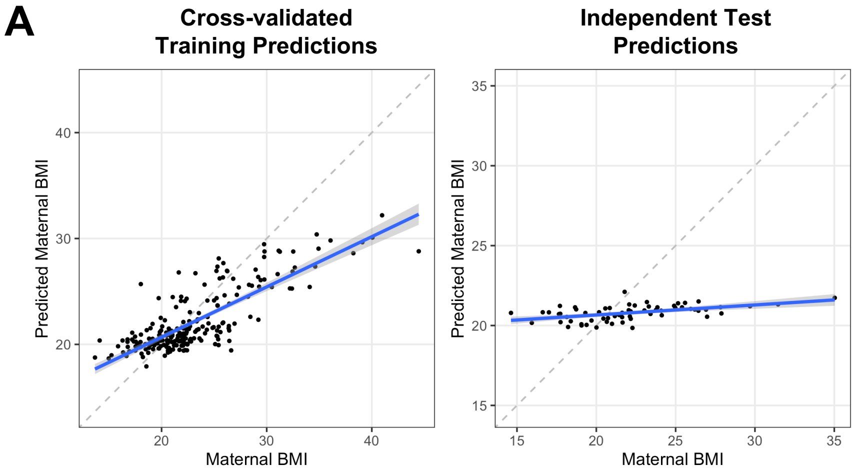


**Figure S12. Validation of the proteomic model of maternal body mass index (BMI).** A cross-validated XGBoost model for the prediction of maternal BMI was trained on the maternal proteome and used to generate predictions in an independent cohort from the AMANHI and GAPPS biorepositories ($N$ = 63). Left: Maternal BMI cross-validated predictions of the proteome model (Pearson's $r$ = 0.81, 95% CI: 0.76-0.85, $P = 1.9*10^{-53}$, RMSE = 3.2, MAE = 2.4, $N$ = 226). Right: Maternal BMI predictions of the proteome model in the independent validation (Pearson's $r$ = 0.50, 95% CI: 0.29-0.67 $P = 12.5*10^{-5}$, RMSE = 3.9, MAE =2.9, $N$ = 63). The blue line and blue shadow represent the regression line and 95% CI of the predicted values versus the ground truth.

**Data S1. (separate file)**

Maternal covariate dictionary

**Data S2. (separate file)**

Metabolomic feature data with extended analyte description

## REFERENCES AND NOTES


1. S. Chawanpaiboon, J. P. Vogel, A.-B. Moller, P. Lumbiganon, M. Petzold, D. Hogan, S. Landoulsi, N. Jampathong, K. Kongwattanakul, M. Laopaiboon, C. Lewis, S. Rattanakanokchai, D. N. Teng, J. Thinkhamrop, K. Watananirun, J. Zhang, W. Zhou, A. M. Gülmezoglu, Global, regional, and national estimates of levels of preterm birth in 2014: A systematic review and modelling analysis. *Lancet Glob. Health* **7**, e37–e46 (2019).

2. S. Saigal, L. W. Doyle, An overview of mortality and sequelae of preterm birth from infancy to adulthood. *Lancet* **371**, 261–269 (2008).

3. J. P. Vogel, S. Chawanpaiboon, A.-B. Moller, K. Watananirun, M. Bonet, P. Lumbiganon, The global epidemiology of preterm birth. *Best Pract. Res. Clin. Obstet. Gynaecol.* **52**, 3–12 (2018).

4. C. Espinosa, M. Becker, I. Marić, R. J. Wong, G. M. Shaw, B. Gaudilliere, N. Aghaeepour, D. K. Stevenson; Prematurity Research Center at Stanford, Data-driven modeling of pregnancy-related complications. *Trends Mol. Med.* **27**, 762–776 (2021).

5. L. S. Peterson, I. A. Stelzer, A. S. Tsai, M. S. Ghaemi, X. Han, K. Ando, V. D. Winn, N. R. Martinez, K. Contrepois, M. N. Moufarrej, S. Quake, D. A. Relman, M. P. Snyder, G. M. Shaw, D. K. Stevenson, R. J. Wong, P. Arck, M. S. Angst, N. Aghaeepour, B. Gaudilliere, Multiomic immune clockworks of pregnancy. *Semin. Immunopathol.* **42**, 397–412 (2020).

6. R. Romero, S. K. Dey, S. J. Fisher, Preterm labor: One syndrome, many causes. *Science* **345**, 760–765 (2014).

7. A. Matei, G. Saccone, J. P. Vogel, A. B. Armson, Primary and secondary prevention of preterm birth: A review of systematic reviews and ongoing randomized controlled trials. *Eur. J. Obstet. Gynecol. Reprod. Biol.* **236**, 224–239 (2019).

8. R. L. Goldenberg, J. F. Culhane, J. D. Iams, R. Romero, Epidemiology and causes of preterm birth. *Lancet* **371**, 75–84 (2008).

9. H. A. Boyd, G. Poulsen, J. Wohlfahrt, J. C. Murray, B. Feenstra, M. Melbye, Maternal contributions to preterm delivery. *Am. J. Epidemiol.* **170**, 1358–1364 (2009).

10. A. Conde-Agudelo, A. Rosas-Bermúdez, A. C. Kafury-Goeta, Birth spacing and risk of adverse perinatal Outcomes. *JAMA* **295**, 1809–1823 (2006).

11. E. Malacova, A. Regan, N. Nassar, C. Raynes-Greenow, H. Leonard, R. Srinivasjois, A. W Shand, T. Lavin, G. Pereira, Risk of stillbirth, preterm delivery, and fetal growth restriction following exposure in a previous birth: Systematic review and meta-analysis. *BJOG* **125**, 183–192 (2018).

12. C. M. Williams, I. Asaolu, N. R. Chavan, L. H. Williamson, A. M. Lewis, L. Beaven, K. B. Ashford, Previous cesarean delivery associated with subsequent preterm birth in the United States. *Eur. J. Obstet. Gynecol. Reprod. Biol.* **229**, 88–93 (2018).

13. R. J. Baer, V. Berghella, L. J. Muglia, M. E. Norton, L. Rand, K. K. Ryckman, L. L. Jelliffe-Pawlowski, M. R. McLemore, Previous adverse outcome of term pregnancy and risk of preterm birth in subsequent pregnancy. *Matern. Child Health J.* **23**, 443–450 (2019).

14. D. K. Stevenson, R. J. Wong, N. Aghaeepour, I. Maric, M. S. Angst, K. Contrepois, G. L. Darmstadt, M. L. Druzin, M. L. Eisenberg, B. Gaudilliere, R. S. Gibbs, I. H. Gotlib, J. B. Gould, H. C. Lee, X. B. Ling, J. A. Mayo, M. N. Moufarrej, C. C. Quaintance, S. R. Quake, D. A. Relman, M. Katz, Towards personalized medicine in maternal and child health: Integrating biologic and social determinants. *Pediatr. Res.* **89**, 252–258 (2021).

15. B. Bekkar, S. Pacheco, R. Basu, N. DeNicola, Association of air pollution and heat exposure with preterm birth, low birth weight, and stillbirth in the US: A systematic review. *JAMA Netw. Open* **3**, e208243 (2020).

16. Y. V. Pusdekar, A. B. Patel, K. G. Kurhe, S. R. Bhargav, V. Thorsten, A. Garces, R. L. Goldenberg, S. S. Goudar, S. Saleem, F. Esamai, E. Chomba, M. Bauserman, C. L. Bose, E. A. Liechty, N. F. Krebs, R. J. Derman, W. A. Carlo, M. Koso-Thomas, T. L. Nolen, E. M. McClure, P. L. Hibberd, Rates and risk factors for preterm birth and low birthweight in the global network sites in six low- and low middle-income countries. *Reprod. Health* **17**, 187 (2020).

17. J. Pervin, S. M. Rahman, M. Rahman, S. Aktar, A. Rahman, Association between antenatal care visit and preterm birth: A cohort study in rural Bangladesh. *BMJ Open* **10**, e036699 (2020).

18. S. Kim, E.-O. Im, J. Liu, C. Ulrich, Maternal age patterns of preterm birth: Exploring the moderating roles of chronic stress and race/ethnicity. *Ann. Behav. Med.* **54**, 653–664 (2020).

19. A. Staneva, F. Bogossian, M. Pritchard, A. Wittkowski, The effects of maternal depression, anxiety, and perceived stress during pregnancy on preterm birth: A systematic review. *Women Birth* **28**, 179–193 (2015).

20. T. A. Manuck, Racial and ethnic differences in preterm birth: A complex, multifactorial problem. *Semin. Perinatol.* **41**, 511–518 (2017).

21. B. Liu, G. Xu, Y. Sun, Y. Du, R. Gao, L. G. Snetselaar, M. K. Santillan, W. Bao, Association between maternal pre-pregnancy obesity and preterm birth according to maternal age and race or ethnicity: A population-based study. *Lancet Diabetes Endocrinol.* **7**, 707–714 (2019).

22. N. Aghaeepour, E. A. Ganio, D. Mcilwain, A. S. Tsai, M. Tingle, S. Van Gassen, D. K. Gaudilliere, Q. Baca, L. McNeil, R. Okada, M. S. Ghaemi, D. Furman, R. J. Wong, V. D. Winn, M. L. Druzin, Y. Y. El-Sayed, C. Quaintance, R. Gibbs, G. L. Darmstadt, G. M. Shaw, D. K. Stevenson, R. Tibshirani, G. P. Nolan, D. B. Lewis, M. S. Angst, B. Gaudilliere, An immune clock of human pregnancy. *Sci. Immunol.* **2**, eaan2946 (2017).

23. N. Aghaeepour, B. Lehallier, Q. Baca, E. A. Ganio, R. J. Wong, M. S. Ghaemi, A. Culos, Y. Y. El-Sayed, Y. J. Blumenfeld, M. L. Druzin, V. D. Winn, R. S. Gibbs, R. Tibshirani, G. M. Shaw, D. K. Stevenson, B. Gaudilliere, M. S. Angst, A proteomic clock of human pregnancy. *Am. J. Obstet. Gynecol.* **218**, 347.e1–347.e14 (2018).

24. L. Liang, M.-L. H. Rasmussen, B. Piening, X. Shen, S. Chen, H. Röst, J. K. Snyder, R. Tibshirani, L. Skotte, N. C. Lee, K. Contrepois, B. Feenstra, H. Zackriah, M. Snyder, M. Melbye, Metabolic dynamics and prediction of gestational age and time to delivery in pregnant women. *Cell* **181**, 1680–1692.e15 (2020).

25. I. A. Stelzer, M. S. Ghaemi, X. Han, K. Ando, J. J. Hédou, D. Feyaerts, L. S. Peterson, K. K. Rumer, E. S. Tsai, E. A. Ganio, D. K. Gaudillière, A. S. Tsai, B. Choisy, L. P. Gaigne, F. Verdonk, D. Jacobsen, S. Gavasso, G. M. Traber, M. Ellenberger, N. Stanley, M. Becker, A. Culos, R. Fallahzadeh, R. J. Wong, G. L. Darmstadt, M. L. Druzin, V. D. Winn, R. S. Gibbs, X. B. Ling, K. Sylvester, B. Carvalho, M. P. Snyder, G. M. Shaw, D. K. Stevenson, K. Contrepois, M. S. Angst, N. Aghaeepour, B. Gaudillière, Integrated trajectories of the maternal metabolome, proteome, and immunome predict labor onset. *Sci. Transl. Med.* **13**, eabd9898 (2021).

26. X. Han, M. S. Ghaemi, K. Ando, L. S. Peterson, E. A. Ganio, A. S. Tsai, D. K. Gaudilliere, I. A. Stelzer, J. Einhaus, B. Bertrand, N. Stanley, A. Culos, A. Tanada, J. Hedou, E. S. Tsai, R. Fallahzadeh, R. J. Wong, A. E. Judy, V. D. Winn, M. L. Druzin, B. Gaudilliere, Differential dynamics of the maternal immune system in healthy pregnancy and preeclampsia. *Front. Immunol.* **10**, 1305 (2019).

27. F. Jehan, S. Sazawal, A. H. Baqui, M. I. Nisar, U. Dhingra, R. Khanam, M. Ilyas, A. Dutta, D. K. Mitra, U. Mehmood, S. Deb, A. Mahmud, A. Hotwani, S. M. Ali, S. Rahman, A. Nizar, S. M. Ame, M. I. Moin, S. Muhammad, A. Chauhan; Alliance for Maternal and Newborn Health Improvement, the Global Alliance to Prevent Prematurity and Stillbirth; and the Prematurity Research Center at Stanford University, Multiomics characterization of preterm birth in low- and middle-income countries. *JAMA Netw. Open* **3**, e2029655 (2020).

28. I. Marić, K. Contrepois, M. N. Moufarrej, I. A. Stelzer, D. Feyaerts, X. Han, A. Tang, N. Stanley, R. J. Wong, G. M. Traber, M. Ellenberger, A. L. Chang, R. Fallahzadeh, H. Nassar, M. Becker, M. Xenochristou, C. Espinosa, D. De Francesco, M. S. Ghaemi, E. K. Costello, A. Culos, X. B. Ling, K. G. Sylvester, G. L. Darmstadt, V. D. Winn, G. M. Shaw, D. A. Relman, S. R. Quake, M. S. Angst, M. P. Snyder, D. K. Stevenson, B. Gaudilliere, N. Aghaeepour, Early prediction and longitudinal modeling of preeclampsia from multiomics. *Patterns* **3**, 100655 (2022).

29. M. S. Ghaemi, A. L. Tarca, R. Romero, N. Stanley, R. Fallahzadeh, A. Tanada, A. Culos, K. Ando, X. Han, Y. J. Blumenfeld, M. L. Druzin, Y. Y. El-Sayed, R. S. Gibbs, V. D. Winn, K. Contrepois, X. B. Ling, R. J. Wong, G. M. Shaw, D. K. Stevenson, B. Gaudilliere, M. S. Angst, Proteomic signatures predict preeclampsia in individual cohorts but not across cohorts - implications for clinical biomarker studies. *J. Matern. Fetal Neonatal Med.* **35**, 5621–5628 (2022).

30. E. Becht, L. McInnes, J. Healy, C.-A. Dutertre, I. W. H. Kwok, L. G. Ng, F. Ginhoux, E. W. Newell, Dimensionality reduction for visualizing single-cell data using UMAP. *Nat. Biotechnol.* **37**, 38–44 (2018).

31. A. Alexa, J. Rahnenführer, Gene set enrichment analysis with topGO. R Package version 2.24.0 (2016).

32. M. Ashburner, C. A. Ball, J. A. Blake, D. Botstein, H. Butler, J. M. Cherry, A. P. Davis, K. Dolinski, S. S. Dwight, J. T. Eppig, M. A. Harris, D. P. Hill, L. Issel-Tarver, A. Kasarskis, S. Lewis, J. C. Matese, J. E. Richardson, M. Ringwald, G. M. Rubin, G. Sherlock, Gene Ontology: Tool for the unification of biology. *Nat. Genet.* **25**, 25–29 (2000).

33. S. Li, Y. Park, S. Duraisingham, F. H. Strobel, N. Khan, Q. A. Soltow, D. P. Jones, B. Pulendran, Predicting network activity from high throughput metabolomics. *PLOS Comput. Biol.* **9**, e1003123 (2013).

34. R. L. Goldenberg, J. F. Culhane, J. D. Iams, R. Romero, Preterm birth 1: Epidemiology and causes of preterm birth. *Obstet. Anesth. Dig.* **29**, 6–7 (2009).

35. T. Chen, C. Guestrin, paper presented at Proceedings of the 22nd ACM SIGKDD International Conference on Knowledge Discovery and Data Mining-KDD'16 (ACM, 2016).

36. J. Davis, M. Goadrich, paper presented at Proceedings of the 23rd International Conference on Machine Learning - ICML'06 (ACM, 2006).

37. M. S. Ghaemi, D. B. DiGiulio, K. Contrepois, B. Callahan, T. T. M. Ngo, B. Lee-McMullen, B. Lehallier, A. Robaczewska, D. Mcilwain, Y. Rosenberg-Hasson, R. J. Wong, C. Quaintance, A. Culos, N. Stanley, A. Tanada, A. Tsai, D. Gaudilliere, E. Ganio, X. Han, K. Ando, N. Aghaeepour, Multiomics modeling of the immunome, transcriptome, microbiome, proteome and metabolome adaptations during human pregnancy. *Bioinformatics* **35**, 95–103 (2019).

38. J. M. Morris, J. Totterdell, Y. S. Bin, J. B. Ford, C. L. Roberts, Contribution of maternal age, medical and obstetric history to maternal and perinatal morbidity/mortality for women aged 35 or older. *Aust. N. Z. J. Obstet. Gynaecol.* **58**, 91–97 (2018).

39. K. Borkowski, J. W. Newman, N. Aghaeepour, J. A. Mayo, I. Blazenović, O. Fiehn, D. K. Stevenson, G. M. Shaw, S. L. Carmichael, Mid-gestation serum lipidomic profile associations with spontaneous preterm birth are influenced by body mass index. *PLOS ONE* **15**, e0239115 (2020).

40. G. M. Shaw, P. H. Wise, J. Mayo, S. L. Carmichael, C. Ley, D. J. Lyell, B. Z. Shachar, K. Melsop, C. S. Phibbs, D. K. Stevenson, J. Parsonnet, J. B. Gould; March of Dimes Prematurity Research Center at Stanford University School of Medicine, Maternal prepregnancy body mass index and risk of spontaneous preterm birth. *Paediatr. Perinat. Epidemiol.* **28**, 302–311 (2014).

41. S. Serapio, F. Ahlsson, A. Larsson, T. Kunovac Kallak, Second trimester maternal leptin levels are associated with body mass index and gestational weight gain but not birth weight of the infant. *Horm. Res. Paediatr.* **92**, 106–114 (2020).

42. A. Xu, Y. Wang, J. Y. Xu, D. Stejskal, S. Tam, J. Zhang, N. M. S. Wat, W. K. Wong, K. S. L. Lam, Adipocyte fatty acid-binding protein is a plasma biomarker closely associated with obesity and metabolic syndrome. *Clin. Chem.* **52**, 405–413 (2006).

43. X. Zhang, H. F. Gu, J. Frystyk, S. Efendic, K. Brismar, A. Thorell, Analyses of IGFBP2 DNA methylation and mRNA expression in visceral and subcutaneous adipose tissues of obese subjects. *Growth Horm. IGF Res.* **45**, 31–36 (2019).

44. M. S. Boyne, M. Thame, F. I. Bennett, C. Osmond, J. P. Miell, T. E. Forrester, The relationship among circulating insulin-like growth factor (IGF)-I, IGF-binding proteins-1 and -2, and birth anthropometry: A prospective study. *J. Clin. Endocrinol. Metab.* **88**, 1687–1691 (2003).

45. A. Weber, G. L. Darmstadt, S. Gruber, M. E. Foeller, S. L. Carmichael, D. K. Stevenson, G. M. Shaw, Application of machine-learning to predict early spontaneous preterm birth among nulliparous non-Hispanic black and white women. *Ann. Epidemiol.* **28**, 783–789.e1 (2018).

46. S. Park, D. Oh, H. Heo, G. Lee, S. M. Kim, A. Ansari, Y.-A. You, Y. J. Jung, Y.-H. Kim, M. Lee, Y. J. Kim, Prediction of preterm birth based on machine learning using bacterial risk score in cervicovaginal fluid. *Am. J. Reprod. Immunol.* **86**, e13435 (2021).

47. C. Gao, S. Osmundson, D. R. Velez Edwards, G. P. Jackson, B. A. Malin, Y. Chen, Deep learning predicts extreme preterm birth from electronic health records. *J. Biomed. Inform.* **100**, 103334 (2019).

48. A. Esty, M. Frize, J. Gilchrist, E. Bariciak, Applying data preprocessing methods to predict premature birth. *Annu. Int. Conf. IEEE Eng. Med. Biol. Soc.* **2018**, 6096–6099 (2018).

49. R. Arabi Belaghi, J. Beyene, S. D. McDonald, Prediction of preterm birth in nulliparous women using logistic regression and machine learning. *PLOS ONE* **16**, e0252025 (2021).

50. A. Koivu, M. Sairanen, Predicting risk of stillbirth and preterm pregnancies with machine learning. *Health Inf. Sci. Syst.* **8**, 14 (2020).

51. A. L. Tarca, B. Á. Pataki, R. Romero, M. Sirota, Y. Guan, R. Kutum, N. Gomez-Lopez, B. Done, G. Bhatti, T. Yu, G. Andreoletti, T. Chaiworapongsa; DREAM Preterm Birth Prediction Challenge Consortium, S. S. Hassan, C.-D. Hsu, N. Aghaeepour, G. Stolovitzky, I. Csabai, J. C. Costello, Crowdsourcing assessment of maternal blood multi-omics for predicting gestational age and preterm birth. *Cell Rep. Med.* **2**, 100323 (2021).

52. A. Abraham, B. Le, I. Kosti, P. Straub, D. R. Velez-Edwards, L. K. Davis, J. M. Newton, L. J. Muglia, A. Rokas, C. A. Bejan, M. Sirota, J. A. Capra, Dense phenotyping from electronic health records enables machine learning-based prediction of preterm birth. *BMC Med.* **20**, 333 (2022).

53. J. G. B. Derraik, M. Lundgren, W. S. Cutfield, F. Ahlsson, Maternal height and preterm birth: A study on 192,432 Swedish women. *PLOS ONE* **11**, e0154304 (2016).

54. Z. Han, S. Mulla, J. Beyene, G. Liao, S. D. McDonald; Knowledge Synthesis Group, Maternal underweight and the risk of preterm birth and low birth weight: A systematic review and meta-analyses. *Int. J. Epidemiol.* **40**, 65–101 (2011).

55. H. Berger, N. Melamed, B. M. Davis, H. Hasan, K. Mawjee, J. Barrett, S. D. McDonald, M. Geary, J. G. Ray, Impact of diabetes, obesity and hypertension on preterm birth: Population-based study. *PLOS ONE* **15**, e0228743 (2020).

56. A. N. Battarbee, R. G. Sinkey, L. M. Harper, S. Oparil, A. T. N. Tita, Chronic hypertension in pregnancy. *Am. J. Obstet. Gynecol.* **222**, 532–541 (2020).

57. A. Cantarutti, M. Franchi, M. Monzio Compagnoni, L. Merlino, G. Corrao, Mother's education and the risk of several neonatal outcomes: An evidence from an Italian population-based study. *BMC Pregnancy Childbirth* **17**, 221 (2017).

58. M. Ruiz, P. Goldblatt, J. Morrison, L. Kukla, J. Švancara, M. Riitta-Järvelin, A. Taanila, M.-J. Saurel-Cubizolles, S. Lioret, C. Bakoula, A. Veltsista, D. Porta, F. Forastiere, M. van Eijsden, T. G. M. Vrijkotte, M. Eggesbø, R. A. White, H. Barros, S. Correia, M. Vrijheid, H. Pikhart, Mother's education and the risk of preterm and small for gestational age birth: A DRIVERS meta-analysis of 12 European cohorts. *J. Epidemiol. Community Health* **69**, 826–833 (2015).

59. T. T. M. Ngo, M. N. Moufarrej, M.-L. H. Rasmussen, J. Camunas-Soler, W. Pan, J. Okamoto, N. F. Neff, K. Liu, R. J. Wong, K. Downes, R. Tibshirani, G. M. Shaw, L. Skotte, D. K. Stevenson, J. R. Biggio, M. A. Elovitz, M. Melbye, S. R. Quake, Noninvasive blood tests for fetal development predict gestational age and preterm delivery. *Science* **360**, 1133–1136 (2018).

60. H. Schock, A. Zeleniuch-Jacquotte, E. Lundin, K. Grankvist, H.-Å. Lakso, A. Idahl, M. Lehtinen, H.-M. Surcel, R. T. Fortner, Hormone concentrations throughout uncomplicated pregnancies: A longitudinal study. *BMC Pregnancy Childbirth* **16**, 146 (2016).

61. L. S. Bleker, T. J. Roseboom, T. G. Vrijkotte, R. M. Reynolds, S. R. de Rooij, Determinants of cortisol during pregnancy—The ABCD cohort. *Psychoneuroendocrinology* **83**, 172–181 (2017).

62. L. Schiffer, L. Barnard, E. S. Baranowski, L. C. Gilligan, A. E. Taylor, W. Arlt, C. H. L. Shackleton, K.-H. Storbeck, Human steroid biosynthesis, metabolism and excretion are differentially reflected by serum and urine steroid metabolomes: A comprehensive review. *J. Steroid Biochem. Mol. Biol.* **194**, 105439 (2019).

63. T. J. Kaitu'u-Lino, S. Tong, S. P. Walker, T. M. MacDonald, P. Cannon, T.-V. Nguyen, S. A. Sadananthan, M.-T. Tint, Y. Y. Ong, L. S. Ling, P. D. Gluckman, Y.-S. Chong, K. M. Godfrey, S.-Y. Chan, K. H. Tan, Y. S. Lee, N. Michael, J. G. Eriksson, M. E. Wlodek, Maternal circulating SPINT1 is

reduced in small-for-gestational age pregnancies at 26 weeks: Growing up in Singapore towards health outcomes (GUSTO) cohort study. *Placenta* **110**, 24–28 (2021).

64. T. J. Kaitu'u-Lino, T. M. MacDonald, P. Cannon, T.-V. Nguyen, R. J. Hiscock, N. Haan, J. E. Myers, R. Hastie, K. M. Dane, A. L. Middleton, I. Bittar, A. N. Sferruzzi-Perri, N. Pritchard, A. Harper, N. J. Hannan, V. Kyritsis, N. Crinis, L. Hui, S. P. Walker, S. Tong, Circulating SPINT1 is a biomarker of pregnancies with poor placental function and fetal growth restriction. *Nat. Commun.* **11**, 2411 (2020).

65. Y.-H. Zhang, M. Tian, M.-X. Tang, Z.-Z. Liu, A.-H. Liao, Recent insight into the role of the PD-1/PD-L1 pathway in feto-maternal tolerance and pregnancy. *Am. J. Reprod. Immunol.* **74**, 201–208 (2015).

66. J. M. Kinder, L. H. Turner, I. A. Stelzer, H. Miller-Handley, A. Burg, T.-Y. Shao, G. Pham, S. S. Way, $CD8^+$ T cell functional exhaustion overrides pregnancy-induced fetal antigen alloimmunization. *Cell Rep.* **31**, 107784 (2020).

67. N. A. Nicola, J. J. Babon, Leukemia inhibitory factor (LIF). *Cytokine Growth Factor Rev.* **26**, 533–544 (2015).

68. T. Mohan, L. Deng, B.-Z. Wang, CCL28 chemokine: An anchoring point bridging innate and adaptive immunity. *Int. Immunopharmacol.* **51**, 165–170 (2017).

69. E. A. L. Enninga, S. M. Harrington, D. J. Creedon, R. Ruano, S. N. Markovic, H. Dong, R. S. Dronca, Immune checkpoint molecules soluble program death ligand 1 and galectin-9 are increased in pregnancy. *Am. J. Reprod. Immunol.* **79**, e12795 (2018).

70. E. Bränn, Å. Edvinsson, A. Rostedt Punga, I. Sundström-Poromaa, A. Skalkidou, Inflammatory and anti-inflammatory markers in plasma: From late pregnancy to early postpartum. *Sci. Rep.* **9**, 1863 (2019).

71. C.-L. Nhan-Chang, R. Romero, J. P. Kusanovic, F. Gotsch, S. S. Edwin, O. Erez, P. Mittal, C. J. Kim, M. J. Kim, J. Espinoza, L. A. Friel, E. Vaisbuch, N. G. Than, S. Mazaki-Tovi, S. S. Hassan, A role for CXCL13 (BCA-1) in pregnancy and intra-amniotic infection/inflammation. *J. Matern. Fetal Neonatal Med.* **21**, 763–775 (2008).

72. Y. A. Barbitoff, A. A. Tsarev, E. S. Vashukova, E. M. Maksiutenko, L. V. Kovalenko, L. D. Belotserkovtseva, A. S. Glotov, A data-driven review of the genetic factors of pregnancy complications. *Int. J. Mol. Sci.* **21**, 3384 (2020).

73. C. Motta-Mejia, N. Kandzija, W. Zhang, V. Mhlomi, A. S. Cerdeira, A. Burdujan, D. Tannetta, R. Dragovic, I. L. Sargent, C. W. Redman, U. Kishore, M. Vatish, Placental vesicles carry active endothelial nitric oxide synthase and their activity is reduced in preeclampsia. *Hypertension* **70**, 372–381 (2017).

74. H. S. Carlsen, E. S. Baekkevold, H. C. Morton, G. Haraldsen, P. Brandtzaeg, Monocyte-like and mature macrophages produce CXCL13 (B cell-attracting chemokine 1) in inflammatory lesions with lymphoid neogenesis. *Blood* **104**, 3021–3027 (2004).

75. R. Muñoz-Fernández, A. Prados, E. Leno-Durán, A. Blázquez, J. R. García-Fernández, G. Ortiz-Ferrón, E. G. Olivares, Human decidual stromal cells secrete C-X-C motif chemokine 13, express B cell-activating factor and rescue B lymphocytes from apoptosis: Distinctive characteristics of follicular dendritic cells. *Hum. Reprod.* **27**, 2775–2784 (2012).

76. B. J. Krause, M. A. Hanson, P. Casanello, Role of nitric oxide in placental vascular development and function. *Placenta* **32**, 797–805 (2011).

77. E. J. Gregory, J. Liu, H. Miller-Handley, J. M. Kinder, S. S. Way, Epidemiology of pregnancy complications through the lens of immunological memory. *Front. Immunol.* **12**, 693189 (2021).

78. B. Koullali, M. D. van Zijl, B. M. Kazemier, M. A. Oudijk, B. W. J. Mol, E. Pajkrt, A. C. J. Ravelli, The association between parity and spontaneous preterm birth: A population based study. *BMC Pregnancy Childbirth* **20**, 233 (2020).

79. D. Goldman-Wohl, M. Gamliel, O. Mandelboim, S. Yagel, Learning from experience: Cellular and molecular bases for improved outcome in subsequent pregnancies. *Am. J. Obstet. Gynecol.* **221**, 183–193 (2019).

80. T. E. C. Kieffer, M. M. Faas, S. A. Scherjon, J. R. Prins, Pregnancy persistently affects memory T cell populations. *J. Reprod. Immunol.* **119**, 1–8 (2017).

81. H. E. Tawfik, J. Cena, R. Schulz, S. Kaufman, Role of oxidative stress in multiparity-induced endothelial dysfunction. *Am. J. Physiol. Heart Circ. Physiol.* **295**, H1736–H1742 (2008).

82. F. Sharifzadeh, M. Kashanian, S. Jouhari, N. Sheikhansari, Relationship between pre-pregnancy maternal BMI with spontaneous preterm delivery and birth weight. *J. Obstet. Gynaecol.* **35**, 354–357 (2015).

83. M. Fasshauer, M. Blüher, M. Stumvoll, Adipokines in gestational diabetes. *Lancet Diabetes Endocrinol.* **2**, 488–499 (2014).

84. H. Salem, T. Rosenfeld, G. Altarescu, S. Grisaru-Granovsky, R. Birk, Maternal and neonatal leptin and leptin receptor polymorphisms associated with preterm birth. *Gene* **591**, 209–213 (2016).

85. H. M. Brockway, S. G. Kallapur, I. A. Buhimschi, C. S. Buhimschi, W. E. Ackerman, L. J. Muglia, H. N. Jones, Unique transcriptomic landscapes identified in idiopathic spontaneous and infection related preterm births compared to normal term births. *PLOS ONE* **14**, e0225062 (2019).

86. K. Contrepois, L. Jiang, M. Snyder, Optimized analytical procedures for the untargeted metabolomic profiling of human urine and plasma by combining hydrophilic interaction (HILIC) and reverse-phase liquid chromatography (RPLC)-mass spectrometry. *Mol. Cell. Proteomics* **14**, 1684–1695 (2015).

87. K. Contrepois, S. Mahmoudi, B. K. Ubhi, K. Papsdorf, D. Hornburg, A. Brunet, M. Snyder, Cross-platform comparison of untargeted and targeted lipidomics approaches on aging mouse plasma. *Sci. Rep.* **8**, 17747 (2018).

88. E. Assarsson, M. Lundberg, G. Holmquist, J. Björkesten, S. B. Thorsen, D. Ekman, A. Eriksson, E. Rennel Dickens, S. Ohlsson, G. Edfeldt, A.-C. Andersson, P. Lindstedt, J. Stenvang, M. Gullberg, S. Fredriksson, Homogenous 96-plex PEA immunoassay exhibiting high sensitivity, specificity, and excellent scalability. *PLOS ONE* **9**, e95192 (2014).

89. Z. Gu, L. Gu, R. Eils, M. Schlesner, B. Brors, Circlize Implements and enhances circular visualization in R. *Bioinformatics* **30**, 2811–2812 (2014).

90. T. M. J. Fruchterman, E. M. Reingold, Graph drawing by force-directed placement. *Softw. Pract. Exper.* **21**, 1129–1164 (1991).

91. M. C. Castillo, N. M. Fuseini, K. Rittenhouse, J. T. Price, B. L. Freeman, H. Mwape, J. Winston, N. Sindano, C. Baruch-Gravett, B. H. Chi, M. P. Kasaro, J. A. Litch, J. S. A. Stringer, B. Vwalika, The Zambian Preterm Birth Prevention Study (ZAPPS): Cohort characteristics at enrollment. *Gates Open Res.* **2**, 25 (2018).

92. AMANHI (Alliance for Maternal and Newborn Health Improvement) Bio–banking Study group), A. H. Baqui, R. Khanam, M. S. Rahman, A. Ahmed, H. H. Rahman, M. I. Moin, S. Ahmed, F. Jehan, I. Nisar, A. Hussain, M. Ilyas, A. Hotwani, M. Sajid, S. Qureshi, A. Zaidi, S. Sazawal, S. M. Ali, S. Deb, M. H. Juma, R. Bahl, Understanding biological mechanisms underlying adverse birth outcomes in developing countries: Protocol for a prospective cohort (AMANHI bio-banking) study. *J. Glob. Health.* **7**, 021202 (2017).

93. M. Bawadekar, D. Shim, C. J. Johnson, T. F. Warner, R. Rebernick, D. Damgaard, C. H. Nielsen, G. J. M. Pruijn, J. E. Nett, M. A. Shelef, Peptidylarginine deiminase 2 is required for tumor necrosis factor alpha-induced citrullination and arthritis, but not neutrophil extracellular trap formation. *J. Autoimmun.* **80**, 39–47 (2017).

94. S. Fan, T. Kind, T. Cajka, S. L. Hazen, W. H. W. Tang, R. Kaddurah-Daouk, M. R. Irvin, D. K. Arnett, D. K. Barupal, O. Fiehn, Systematic error removal using random forest for normalizing large-scale untargeted lipidomics data. *Anal. Chem.* **91**, 3590–3596 (2019).

95. P. S. Arunachalam, F. Wimmers, C. K. P. Mok, R. A. P. M. Perera, M. Scott, T. Hagan, N. Sigal, Y. Feng, L. Bristow, O. Tak-Yin Tsang, D. Wagh, J. Coller, K. L. Pellegrini, D. Kazmin, G. Alaaeddine, W. S. Leung, J. M. C. Chan, T. S. H. Chik, C. Y. C. Choi, C. Huerta, B. Pulendran, Systems biological assessment of immunity to mild versus severe COVID-19 infection in humans. *Science* **369**, 1210–1220 (2020).

96. B. Jónsdóttir, M. Ziebell Severinsen, F. von Wowern, C. San Miguel, J. P. Goetze, O. Melander, ST2 predicts mortality in patients with acute hypercapnic respiratory failure treated with noninvasive positive pressure ventilation. *Int. J. Chron. Obstruct. Pulmon. Dis.* **14**, 2385–2393 (2019).

97. A. Tancin Lambert, X. Y. Kong, B. Ratajczak-Tretel, D. Atar, D. Russell, M. Skjelland, V. Bjerkeli, K. Skagen, M. Coq, E. Schordan, H. Firat, B. Halvorsen, A. H. Aamodt, Biomarkers associated with atrial fibrillation in patients with ischemic stroke: A pilot study from the NOR-FIB study. *Cerebrovasc. Dis. Extra* **10**, 11–20 (2020).
